# Improved modeling of RNA-binding protein motifs in an interpretable neural model of RNA splicing

**DOI:** 10.1186/s13059-023-03162-x

**Published:** 2024-01-16

**Authors:** Kavi Gupta, Chenxi Yang, Kayla McCue, Osbert Bastani, Phillip A. Sharp, Christopher B. Burge, Armando Solar-Lezama

**Affiliations:** 1 Department of Electrical Engineering and Computer Science, Massachusetts Institute of Technology, Cambridge, MA 02139 USA; 2 Department of Computer Science, University of Texas at Austin, Austin, TX 78712 USA; 3 Department of Biology, Massachusetts Institute of Technology, Cambridge, MA 02139 USA; 4 Department of Computer and Information Science, University of Pennsylvania, Philadelphia, PA 19104 USA; 5 Koch Institute of Integrative Cancer Research, Massachusetts Institute of Technology, Cambridge, MA 02139 USA

**Keywords:** Alternative splicing, Genome interpretation, Machine learning, Neural network, RNA processing, RNA-binding protein, Variant interpretation

## Abstract

Sequence-specific RNA-binding proteins (RBPs) play central roles in splicing decisions. Here, we describe a modular splicing architecture that leverages in vitro-derived RNA affinity models for 79 human RBPs and the annotated human genome to produce improved models of RBP binding and activity. Binding and activity are modeled by separate Motif and Aggregator components that can be mixed and matched, enforcing sparsity to improve interpretability. Training a new Adjusted Motif (AM) architecture on the splicing task not only yields better splicing predictions but also improves prediction of RBP-binding sites in vivo and of splicing activity, assessed using independent data.

**Supplementary Information:**

The online version contains supplementary material available at 10.1186/s13059-023-03162-x.

## Background

Genomic sequences encode the form and function of organisms, and their interpretation is an important scientific goal. The complex gene architectures present in metazoans makes this particularly challenging. Non-coding segments, or introns, frequently interrupt the coding sequence in eukaryotic genes (with 10 or more per gene on average in mammals), requiring intron excision and ligation of the flanking exons by the RNA splicing machinery in order to assemble the mature protein-coding mRNA [1]. Thus, finding the boundary elements or splice sites that are recognized in this process, the RNA splicing code, is important in interpreting the genome, and understanding how these sites are recognized by the spliceosome is a longstanding puzzle in molecular biology.

**Fig. 1:**
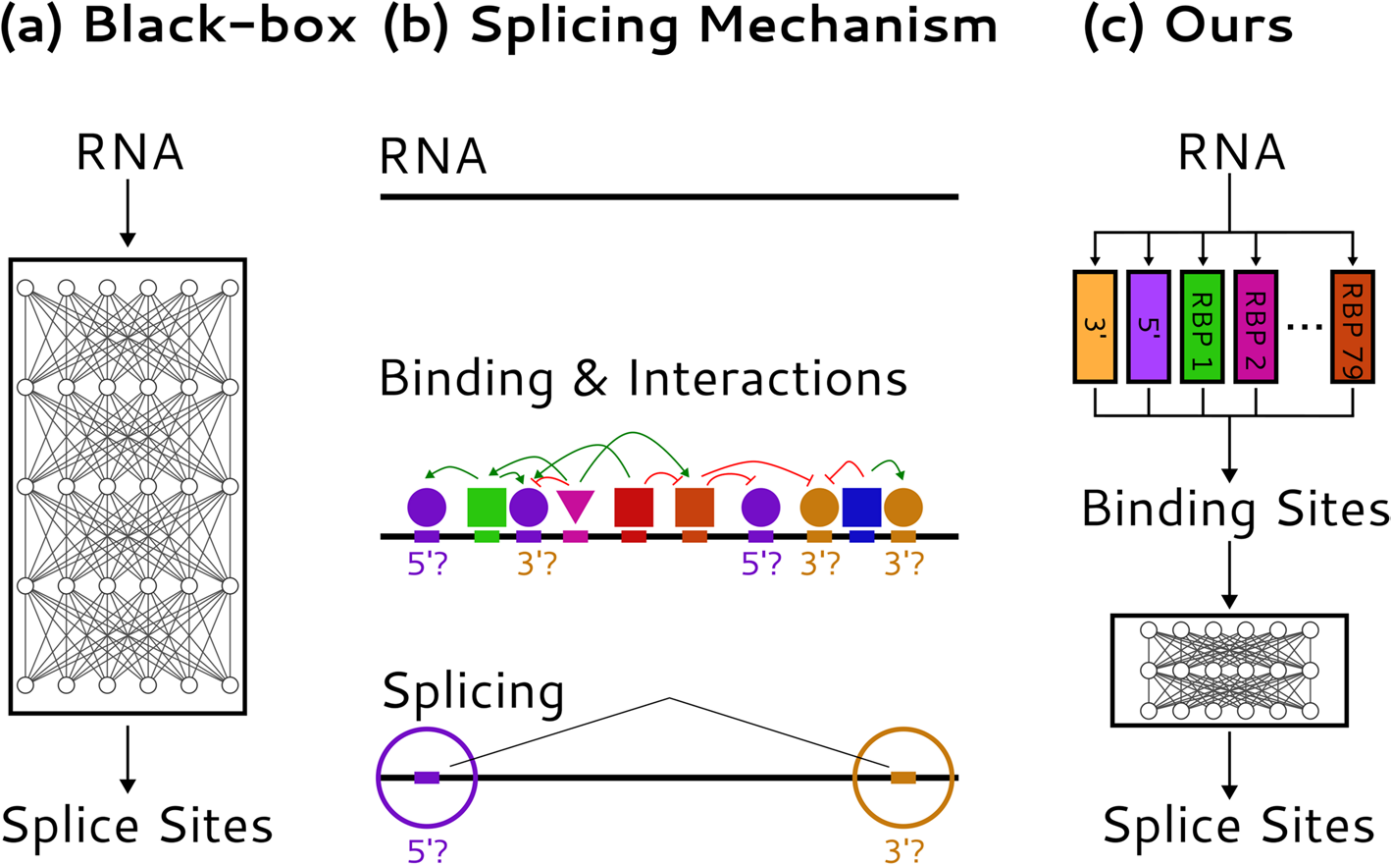
Types of models of RNA splicing. **a** SpliceAI models splicing as a black box mapping from pre-mRNA to splice sites. **b** We conceptualize splicing as a process in which various protein and ribonucleoprotein factors bind to the pre-mRNA sequence and influence which sites are chosen as splice sites and which are not. **c** Our SAM model predicts 5′ and 3′ splice site motifs and RBP binding motifs first, and then predicts which splice sites will be chosen by modeling interactions between these motifs

As the human genome was being sequenced and assembled, gene prediction was a priority, and incorporating detailed models of RNA splicing features proved useful [2]. Models more specifically focused on various aspects of splicing rather than gene finding have since been developed. The splice site motifs were the first aspects to be modeled, typically by giving scores for short sequences as potential 5′ splice sites (5′SS) or 3′ splice sites (3′SS)—the sites recognized at the beginning and ends of introns [3–5]. However, these motifs were found to be insufficient to predict splicing patterns, even in the simpler case where all introns are short [6], implying that other features must broadly contribute to intron recognition. Subsequent efforts have focused on splicing regulatory elements (SREs)—short RNA segments that typically function by recruiting splicing regulatory factors (SRFs), RBPs that promote or inhibit assembly of core machinery at nearby splice sites [1] (Fig. 1). Incorporating known exonic SREs into a simple splicing model was found to substantially improve its predictions [7].

Related problems have since been tackled, such as predicting the splicing phenotypes of mutations or predicting aspects of alternative splicing such as the percent spliced in (PSI) values of exons or the direction of changes in exon inclusion between different tissues [8–13]. Most models emphasized features known to be recognized in splicing, but to improve accuracy, some models also considered features not available to the spliceosome, such as evolutionary conservation [14, 15].

More recently, some attention has returned to the question of predicting splicing from raw sequence using neural network methods that have been highly successful at predictive tasks in many fields [16–18]. Rather than curating features by hand, these machine learning methods have feature selection built into their training process, albeit in a manner that makes extracting and interpreting these features extremely difficult. For instance, SpliceAI, a deep convolutional neural network (CNN), can produce extremely high end-to-end accuracy in predicting splice sites in the human genome using up to 10,000 bases of sequence context. The model is essentially a black box that does not allow extraction of which specific features are of general utility in predicting splicing, although some clues can be gleaned about sequences important for prediction of individual splice sites using in silico mutagenesis [18]. Related CNNs have been developed for prediction of tissue-specific splicing patterns [19, 20]. Some recent work [21] has explored more interpretable learned models of splicing but has done so in the domain of short (70 nt) synthetic exons rather than gene-level splicing.

A recent line of research in AI has focused on interpretable neural networks that use some intermediate processing of the input that corresponds to known information about a particular process [22, 23]. These techniques use auxiliary losses on intermediate layers to ensure that the network actually makes predictions based on general underlying data. Here, we instead use a hard constraint that requires the intermediate concept to resemble previously known information, which is made possible using a sparsity-based approach. Other techniques use multiple datasets with the same intermediate features [24], which is unfortunately not applicable to the splicing domain. Yet other techniques attempt to disentangle features of the input, in such a way as to extract information about the intermediate state [25]; however, these techniques attempt to preserve all information in the input, while we wish to force the model to use only a limited set of well-defined motifs and binding locations.

Here, we sought to develop predictive splicing models that are interpretable and achieve high accuracy on gene-level splicing, with interpretability the paramount goal. This was achieved by developing sub-models inside of our Sparse Adjusted Motif (SAM) model that are directly anchored to known biology. Our Local Splice Site Identifier (LSSI) model learns the 3′SS and 5′SS motifs, and what we call the Fixed Motif (FM) model estimates the binding locations of RBPs using existing biophysically based models derived from in vitro RBP:RNA binding data [26, 27]. These two models, which each predict the binding sites of some protein, have their outputs fed into an Aggregator model, which is then used to predict the actual splicing outcomes. This two-step process ensures that we predict splicing from a known intermediate value, rather than from uninterpretable latent features learned directly from the RNA sequence. We further developed an Adjusted Motif (AM) model which tunes these motifs as part of end-to-end training on the splicing task. This approach affords a substantial increase in accuracy while ensuring that the AM models describe in vitro RBP binding comparably well as the original FM models, preserving the ability to infer the involvement of specific motifs and associated RBPs in splicing and to interpret variants that alter RBP binding.

## Results and discussion

### A modular architecture for splicing with enforced sparsity

**Fig. 2:**
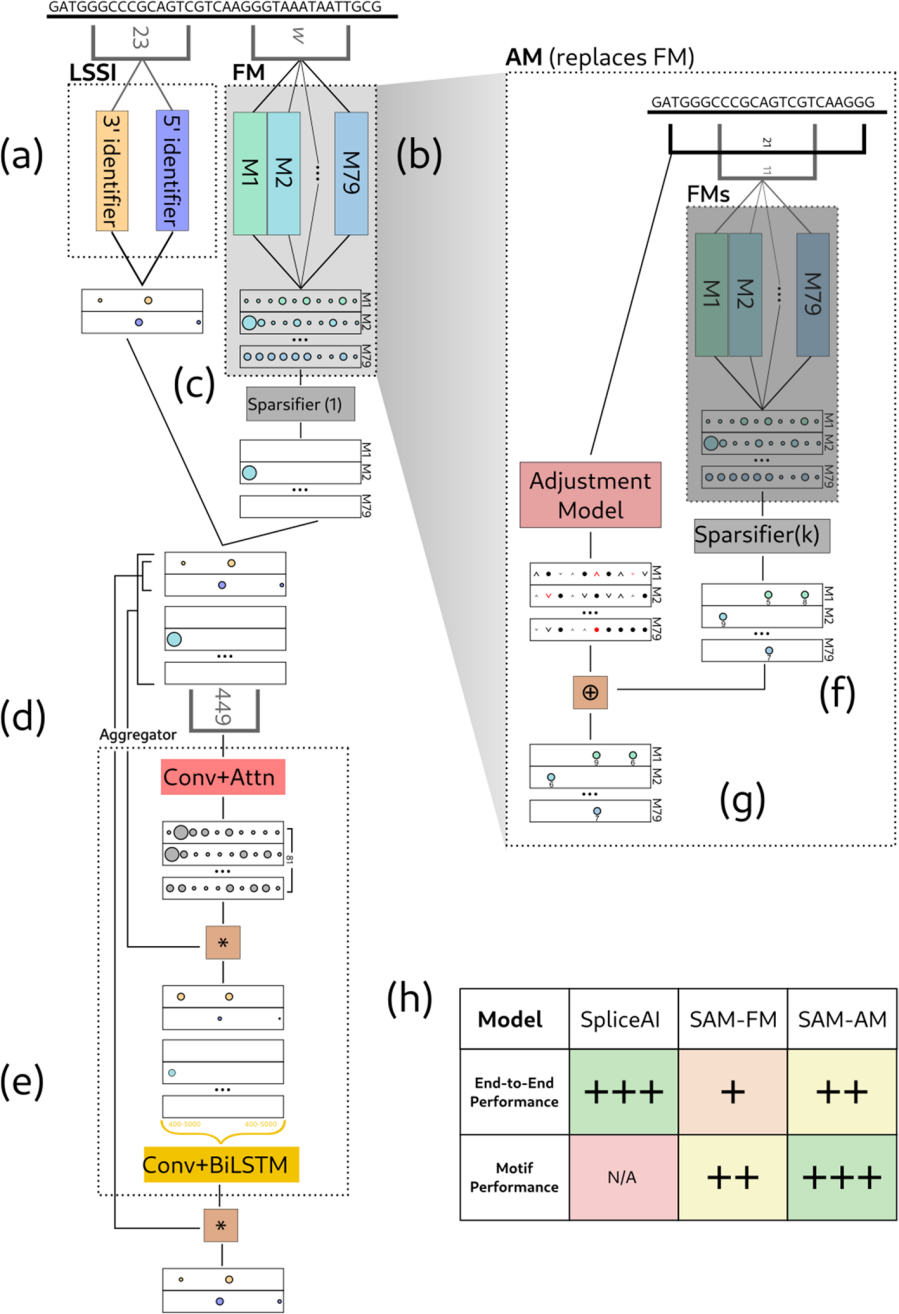
Overview of Sparse Adjusted Motif architecture: components and flow of information. Panels **a** through **e** summarize our splicing architecture using the baseline RBNS PSAM models (FM model); panels **d** and **e** describe the “Aggregator” component of our model, inspired by [28]; panels **f** and **g** summarize training of the “AM” models, which then replace the FM motifs shown in panel b. **a** The LSSI model processes the sequence and produces an annotation of the core 3′ and 5′ motifs. **b** The motif model processes the sequence and produces an estimate of RBP binding affinity at each site. **c** We enforce sparsity on the motif binding affinities, only allowing through high-affinity sites. **d** We compute influence scores for each position in the sequence; by multiplying with the sparse input, we ensure that these influence values are only used to increase or decrease the strengths of known binding sites. **e** We then run a long-range processor across the sequence to score potential splice sites. We multiply these with the outputs of the core motifs to produce our final predictions. We use an LSTM here as the structure of an LSTM’s dataflow graph is identical to that of the forward-backward algorithm, which is commonly used to find the marginal probabilities of states in an HSMM. **f** In our AM model, we first run our FM model and then sparsify it to a level of density *k* times denser than we intend to output (typically *k* = 2). **g** We also predict increase/decrease scores at each position. These scores are then added only to sites that were plausible binding sites. We then resparsify the output. This allows changing both the magnitudes of the sites arbitrarily as well as changing which sites are selected, while guaranteeing that all the produced AM sites are among the sites scored highly by the FM. Specifically, we compute AM’s output as AM(*x*) = FM(*x*) ⊕ Adj(*x*), where *u* ⊕ *v* = 1(*u* ≠ 0)(*u* + *v*). **h** Table represents a high-level view of the two metrics of accuracy we consider. Both the FM and AM models are novel, though under the FM model, the Aggregator is novel while the motifs are previously described [27]. Our AM model improves over the FM model in all features, trading some accuracy versus SpliceAI in favor of being able to predict relevant RBP binding positions, and is the best at binding motif prediction. N/A indicates that SpliceAI is not capable of binding motif prediction

Our approach combines neural techniques with a modular structure that constrains the model to consider elements relevant to splicing. The three main model components are (1) a Local Splice Site Identifier (LSSI) which models the 5′SS and polypyrimidine tract (PPT)/3′SS; (2) a “Motif Model,” which incorporates motifs representing in vitro binding specificity of several dozen RBPs; and (3) an “Aggregator,” which predicts the locations of splice sites based on the LSSI and Motif Model outputs (Fig. 2).

The 3′SS and 5′SS have fairly strong core motifs, which are often captured by graphical models trained using maximum-entropy estimation or other methods [5]. We chose to model the same core splice site regions modeled by the MaxEnt method to facilitate comparison. The 3′SS region comprises 23 nt, consisting of the last 20 bases of the intron and first 3 bases of the succeeding exon, while the 5′SS region comprises 9 nt, consisting of the last 3 bases of the exon and first 6 bases of the downstream intron. In general, our LSSI models slightly outperform MaxEnt models, with top-*k* accuracies for 3′SS and 5′SS on the SpliceAI test set of 23.5% and 26.5% versus 18.4% and 24.7% for the MaxEnt model (Top-k accuracy is the fraction of splice sites predicted correctly at a score cutoff which predicts the same number of splice sites as actually appear in the data).

Next, we considered models of RBP-binding sites in the Motif Model. In our baseline model, we use what we call Fixed Motifs (FMs), which consist of position-specific affinity matrices (PSAMs) derived by the RBPAmp algorithm [27] from RNA Bind-n-Seq (RBNS) in vitro data for 79 human RBPs [26]. Each RBP is represented by 1–5 PSAMs (typically ~11 nt long) that yield relative affinity values for any RNA sequence. We have also explored using PSAM models inferred from RNACompete data [29]. To score a sequence with the PSAM(s) for an RBP, we compute relative affinity from the matrix, then multiply this value by the corresponding absolute affinity, and sum the affinities across all PSAMs. Our primary contribution, the AM Model described below, is an alternative to the PSAM-based FM model. In this paper, the FM model is treated as a baseline as it represents a high-quality model of RBP binding from prior literature.

One consideration in designing a modular neural network structure is to ensure that the LSSI and Motif model outputs are not simply used by the Aggregator to reconstruct the original sequence and then learn a black box splicing algorithm. If this were possible, high accuracy might be achieved without the essential properties of modularity and interpretability. Therefore, it is important to ensure that these models compress the information in the sequence, thus preventing it from being reconstructed. Enforcing an information bottleneck in order to ensure modularity is a standard technique in machine learning [30]; we use a recently described approach [31].

For the LSSI model, we ensure sequence compression by enforcing the minimum score to be a log-probability of −10, by setting all values below −10 to −10. This bar, if interpreted as a binary classification bar, has the effect of including almost all splice sites (98.88% of 3′SS, 99.10% of 5′SS) while assigning meaningful (> −10) scores to just a small fraction of locations (2.00% for the 3′SS model, 1.41% for the 5′SS model, a mean of 1.7% of positions). The LSSI model emphasizes recall over precision as one of its functions in the model is as a mask: any position scored −10 by both LSSI models will not be considered as a potential SS subsequently. Since we assign meaningful values to only 1–2% of locations for each splice site type, and this model is trained entirely separately from the main model, it greatly compresses the input sequence and could contribute at most minimally to sequence reconstruction.

### Enforcing motif sparsity

When modeling SRFs, it is more important to precisely bound the mutual information between the input RNA sequence and the motif layer, as there are many RBPs and thus much more information could theoretically pass through. We use the fact that we know that RBPs bind specific sequences and have finite free concentrations, so that only a fraction of sites in the transcriptome are occupied by RBPs. Therefore, we add a layer immediately after our Motif Model that enforces sparsity, limiting the fraction of sites at which the model considers binding by an RBP. We enforce the density, the number of positions that contain a nonzero value, to be at most δ by enforcing that the sparsity is at least (1 − δ). If we have *M* motif models, if each predicted RBP binding score contains at most *η* bits of information, and we analyze *L* bases, we can bound the entropy of the motifs layer by *H/L* ≤ *M[H(B(δ)) + δ η]*, where *B(x)* represents the Bernoulli distribution with probability *x*, *δ* is the mean motif density, and *η* represents an upper bound across channels of the entropy in the distribution of nonzero activations (Additional file 1: Figure S1; derivation provided in Additional file 1).

This quantity represents the information per base that is being let through by the Motif Model. In general, we require *H/L* < 1.91 bits, in order to ensure that the sequence is being compressed. Throughout this paper, we use *M* = 79, which is the number of RBPs for which we used PSAMs from RBNS as input, unless otherwise specified, and we set *δ* = 0.18% to be our definition of sequence compression. This value allows *η* < 2.8 bits while maintaining sequence compression. In general, the outputs of our sparse layer contain less than 2.8 bits each, with typical values of about 2 bits (Additional file 1).

We empirically confirmed that our entropy bound is sound, but not particularly tight, via an experiment where we trained a neural network to reconstruct the sequence. From the motif output of an AM model with *H/L* bound of 1.79b/nt, the original transcript sequence could be reconstructed with only 59% accuracy in one experiment. This corresponds to an effective *H/L* of 1.16 bits/nt. However, we continue to use 0.18% density in order to ensure that the motif density is well below the theoretical limit required to guarantee compression. Enforcing an entropy bound therefore ensures that the algorithm learns a compressed representation of the sequence and cannot reconstruct the input sequence.

To enforce a maximum density of *δ*, we follow the Sparling technique [31], with a minor variation that we find improves accuracy at the cost of training efficiency. To train this model, our “standard training” approach is as follows. We start with a very large *δ* value of 75%. As training progresses, we reduce the maximum density threshold *δ* by a factor of 0.75 at specific steps, allowing the thresholds to update via the moving average. Rather than pick a certain number of training steps between reductions in *δ*, we instead use validation accuracy as a guide. At specific steps *t* (20 times per epoch), we check whether *V(t)* has reached some target accuracy *VT(t)* and, if so, reduce *δ* to 75% of its previous value.

Unless otherwise specified, we choose *VT(t) = VT* to be a constant in *t* and tune the value of *VT* externally. Specifically, we search for the largest *VT* such that we can train our model to achieve a density of *δ* = 0.18%. In some experiments where it was of interest to evaluate the accuracy resulting from various minor model or data changes, we instead use a “quick training” method equivalent to the Sparling approach. In this approach, we set *VT(t)* to a dynamic function that starts at a high value (typically 85%), then reduces by 1% per epoch of training, increasing to whatever validation accuracy was achieved at the previous step whenever δ is reduced. Though faster, the quick training approach tends to produce slightly (~1–2%) lower performance.

We use the same train/test split as SpliceAI, with the following modification: we treat the first 50% of the SpliceAI test set (by total sequence length) as a validation set, which is used to determine when to adjust sparsity/accuracy thresholds. We use the last 45% of the SpliceAI test set as a true test set, for our evaluation results, with the 5% gap serving to avoid overlap of genes between training and validation sets. Different random orders of genes are used in training/testing.

### Aggregator

The final component of SAM is the Aggregator, a network that aggregates potential splice sites and RBP-binding sites identified by the LSSI and Motif Model and produces a prediction of splice site locations. This network is structured in the following way: the LSSI output is concatenated with the Motif model output, and then processed by a small convolutional model of width 49 nt, then an attention layer of width 401 nt, which is then multiplied with the output of the motif model to propagate the sparsity. We use a custom attention layer design that takes advantage of this limited attention region for performance reasons. We then apply another CNN followed by a long-range BiLSTM network, and the result is then multiplied by the output of the LSSI model to produce our final splicing prediction. The design of the Aggregator is inspired by the hierarchical attention network structure [28], and we believe that the two components roughly correspond to RBP influence (attention, which considers pairwise relationships) and 3′SS/5′SS matching (BiLSTM, which has the same structure as an invocation of the forward-backward algorithm on HMMs). However, without the multiplication and convolution (pre-BiLSTM) operations, the model cannot effectively capture the intrinsic importance of the motifs.

We deliberately decided not to focus on or optimize the Aggregator in this paper, mostly treating it as an opaque box, except in a few instances where we use it to infer patterns of motif activity. However, we do believe that there is no obvious way to improve the Aggregator’s performance. We provide an analysis comparing our Aggregator with a SpliceAI-like convolutional network (Additional file 1: Figure S2) and find that, while this change helps with end-to-end performance, it reduces motif performance. Additionally, we investigated several changes involving simplifying the aggregator, and all resulted in reduced end-to-end performance.

### Adjusted motif model

**Fig. 3:**
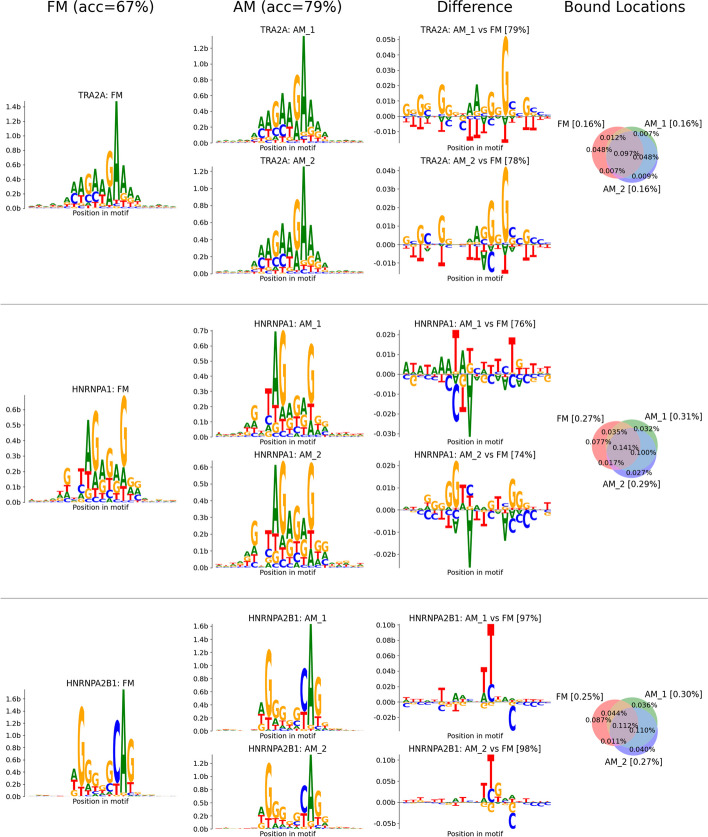
Sequence logos for corresponding AMs and FMs show overall similarity with minor differences. Sequence logos are shown for three representative SRFs: TRA2A, a splicing activator, and splicing repressors HNRNPA1 and HNRNPA2B1. The first column shows the FM motif and the second shows AM logos for 2 replicate runs. The third column shows differences between each AM and the corresponding FM: each base’s height represents its pointwise mutual information, with those above the bar enriched in the AM and those below enriched in the FM. The pattern of differences is often but not always related between replicates, as shown by the examples above. The accuracy a logistic model can achieve at distinguishing AM from FM motifs is shown above each difference plot. The fourth column shows the overlap between FM and AM binding sites for each factor, labeled with the fraction of genomic positions for each category. The total fraction of genomic positions for each model is shown next to the model’s name in square brackets. For example, FMs and AMs for TRA2A bind 0.16% of genomic sites, i.e., one site every 625 nt

We observed that the accuracy achieved with FM models was moderately high (67.2% of splice sites correct, see below for details) but wondered whether these models of in vitro binding of 79 RBPs were optimal for predicting splicing. As an experiment, we allowed the parameters to change subtly during training, while taking steps to preserve the association with individual RBPs, yielding “adjusted motifs” (AMs) (Fig. 3). For example, binding preferences of RBPs in vivo might occasionally differ from the in vitro-derived FM models as a result of binding partners [32] or post-translational modifications [33]. Furthermore, many RBPs belong to families of related proteins, usually with similar but not identical RNA-binding preferences, so the AMs might learn the binding preferences of the SRFs with most important roles in splicing from information about related family members analyzed in vitro. Training an unconstrained neural model to replace the FMs might also improve accuracy, of course, but at the expense of interpretability, as the learned motifs would no longer correspond to known RBPs.

We compute the binding sites in the adjusted motif (AM) model by adjusting the scores for already relatively high-scoring FM binding sites, allowing *k* times as many FM sites as we will eventually use, allowing our AM model to filter out a subset of binding sites among plausible sites provided by the FM model (Methods). Here, we have used *k* = 2: for example, the top 1000 sites scored by an AM model would be constrained to fall within the top 2000 sites scored by the corresponding FM model. This value of *k* serves to constrain the sequence features of AMs to closely resemble the FM motifs in virtually every case, as desired (examples are shown in Fig. 3). Each AM was modeled as a CNN composed of 5 residual units, each with two convolutional layers of width 3, resulting in a total context window of 21 nt. An exploration of motif widths obtained better results with width 21 than with shorter widths (Additional file 1: Figure S3).

We also directly examined the sequence logos corresponding to each motif to assess the adjustments. In general, we found that the motifs tend to be quite similar, with slight differences at a subset of positions (Fig. 3), and modest variation from replicate to replicate. To assess the information captured by the AM models, we trained a logistic classifier with four parameters corresponding to every position in the motif (one-hot encoding of the nucleic acids) to predict the difference between sites classified as bound exclusively by the AM and those classified as bound just by the FM. In general, these classifiers had 75–100% accuracy (average of 90% across RBPs), demonstrating that a large fraction of the differential information captured by the AMs is attributable to linear combinations of the bases present at different motif positions.

As a first evaluation, we evaluated the AM models on the raw RBNS read data used by RBPAmp to fit the RBNS PSAMs. If this result were significantly worse than the FMs (RBNS PSAMs), it would indicate substantial drift from the FMs and thus a failure in the conceptualization of the AM constraint. However, we find that we make a 0.11% *improvement* in accuracy on this task on average (range of −0.02% to +0.25% over 5 seeds), demonstrating that the AMs remain plausible (and even improved) models of RBNS binding.

The substantial improvement in splicing prediction from the AM models, while maintaining discrimination in the in vitro binding task, raised the question of whether neural models specifically trained on the RBNS dataset could improve splicing prediction (see the “Neural models trained on RBNS data” section for more references). The FM models have widths of ~11 nt, but a neural model with width 11nt did not improve discrimination on the RBNS dataset (−0.02% average change in performance), suggesting that the PSAMs are near-optimal representations at width 11. A neural model of width 14 nt and an AM-architecture model (of width 21) trained on RBNS data improved performance on RBNS data by 0.33% and 1.76%, respectively. We refer to the 21-wide AM-architecture model trained on RBNS as a “Neural Fixed Motif” (NFM) model, since the parameters are frozen after training on the in vitro RBNS data and not allowed to vary during training on the splicing task. When the NFM was used in splicing prediction with an adaptive accuracy threshold, performance was 64.1%, slightly below the FM model’s 65.3% and far below the accuracy of 78.6% achieved by AMs trained on the splicing task (these values are lower than those presented earlier as they resulted from the quick training approach). Our interpretation of these observations is that training of AMs on the splicing task using genomic data enables learning of specific sequence features relevant to binding and/or splicing in vivo that are likely not present in the in vitro RBNS data. For example, the AMs might be learning a motif bound in vivo that differs somewhat from the in vitro motif because of the presence of specific post-translational modifications or binding partners of the RBP. Alternatively, instead of the motif of the single RBP analyzed in vitro, the AMs might be learning a motif bound by a close paralog, or a composite motif bound by some or all members of the protein family to which the RBP belongs, and/or other proteins.

As a control, we checked to see to what extent RBNS binding activity correlated with positions that SpliceAI found important. We performed an in silico mutagenesis experiment on SpliceAI, computing an activity score for positions in the sequence as the effect that mutating them had on SpliceAI score. While 3′SS and 5′SS sites clearly had more of an effect than other sites, this was not true of RBP-binding sites as predicted by RBNS. On the other hand, we found that for SAM models, both FM and AM, RBP-binding sites did tend to have greater activity scores (see Additional file 1: Figure S4 for more information). We can thus conclude that SpliceAI is not representing a model of splicing equivalent to ours and is focusing instead on other features, which may or may not correspond to RBPs.

### Module substitution experiment

Our SAM model is designed to be explicitly modular. Setting aside the LSSI for now, which was unchanged during training, our model can effectively be considered as a composition of a Motif Model, *M*, and an Aggregator, *A*. In a traditional neural network, the intermediate layers of a model represent latent variables that are not necessarily stable across different models, as they may converge to different representations of the same information or may even represent different pieces of information relevant to the learned task. However, in our case, we intend M and A to represent the non-latent concepts of RBP-binding sites and the activities of these RBPs in splicing, respectively. If this expectation holds, we should be able to use one model’s *M* with another model’s *A* and still achieve good performance. An alternative, undesirable possibility would be if the AM models side-channel information through their outputs that the Aggregator then learns to pick up, which do not represent activities of the RBPs.

**Fig. 4:**
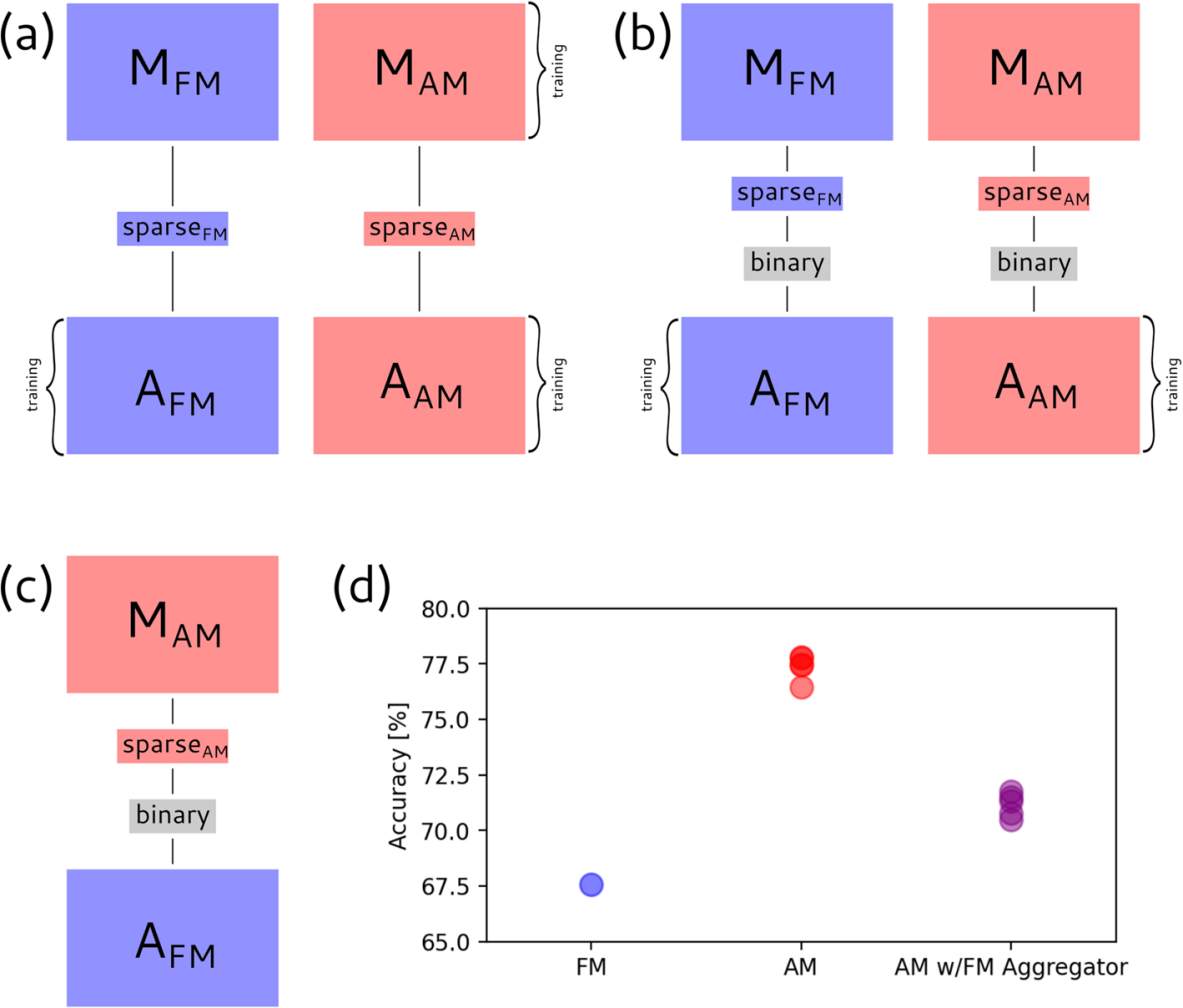
Module substitution shows that Aggregator learns similar SRF activities when trained with FMs or AMs. In this experiment, we demonstrate that the AM model still produces motif sites that carry the same semantic meaning as the original FM sites. To this end, we take the AM motifs and send them to the FM aggregator. If the AM model were producing non-motif information, this would lead to a degradation or at best no change in performance. However, we observed an improvement, from ~68 to ~71% accuracy, demonstrating that the AM models are better at predicting motif binding sites, even provided to a model trained to work with FM predictions. **a**–**c** Stages of the module substitution process. The FM Motif Model and Aggregator are in blue, while those corresponding to the AM model are in red. **a** Both models are trained as usual. **b** The models are binarized by applying a binary layer and then the FM model’s Aggregator is retrained to handle the new binary motif outputs. **c** The new FM Aggregator is used in conjunction with the binarized AM motif model, with sparse layers and binarization layers as shown. **d** Results of the combination experiment

To distinguish between these alternatives, we conducted a computational “Module Substitution” experiment (Fig. 4) in which we use the AM models as the motif model in our architecture and combine it with the Aggregator trained to work with the FMs. One subtlety here is that our training process for AMs cannot guarantee that the actual values of activations are similar across models, so we resolve this by adding a “binarization” layer to pre-trained models that sets all non-zero motif binding site values to 1, followed by retraining of the Aggregator.

The results of this experiment (Fig. 4) reveal two clear trends. Comparing to our previous results (Fig. 3), the AM models tend to perform slightly (1.2–2.9%) worse after binarization, while the FM models are unaffected (Additional file 1: Figure S1 corroborates this, though in the case where the aggregator is not retrained). This observation suggests that the AM models provide information through motif “strength” as well as location, while the FM models’ utility is primarily in identifying potential locations of binding. More importantly, AMs tend to outperform FMs across the board, even when using the FM Aggregator. Since the FM Aggregator was trained without ever seeing AMs, it cannot be side-channeling information unique to the AMs. Instead, the consistent improvement seen with AMs here demonstrates that they largely provide information of the same type (i.e., binding of the same RBPs) as provided by FMs but of improved quality. The further improvement seen when using the AM Aggregator is expected and may reflect that this model makes better use of the motif strength information provided by AMs.

### Analysis of eCLIP binding data

To understand how well the FM and AM models reflect binding conditions in vivo, we used data from the enhanced crosslinking and immunoprecipitation (eCLIP) assay, which has been widely applied to human RBPs in two cell types as part of the ENCODE project [34]. The eCLIP datasets consist of sets of eCLIP peaks, each consisting of a start and end location in the genome that represent the approximate binding location of the associated RBP. At no point were our AM or FM models trained on eCLIP data in any way, so these data provide an independent assessment of the extent to which these models capture RBP binding information. All our analysis results in this section were performed on the subset of 18 RBPs where we had both RBNS PSAMs and eCLIP peak data. We defined “eCLIP regions” by extending the eCLIP peak an additional 50 nt to the 5′ end, since this was found to enrich for known binding motifs [35]. We selected a random sample of 10,000 5 kb regions from the SpliceAI test set for our experiments.

Our metric for eCLIP accuracy is eCLIP enrichment of a model versus the FM model at equivalent sparsity, calculated separately on intronic and exonic sites. It was important to control for intron/exon bias as eCLIP tends to preferentially detect exons, both because some crosslinking may occur in the cytoplasm which is essentially devoid of introns and because even in the nucleus introns have shorter half-lives than exons because they are rapidly degraded after excision. Thus, any bias toward exons could skew the results. Therefore, we present results independently for introns and exons. It was also important to use equivalent sparsity, since enrichment is sensitive to small changes in sparsity (Methods).

As a point of comparison, we also trained AM-format models directly on eCLIP data, designating these models “AM-E.” We did so by setting up a training objective where the model’s output was treated as predicting the probability that each location within a peak is not a match (treating the AM output as the negative log probability of non-match), then multiplying those probabilities together to produce an overall probability of non-match in each peak, and then using the logistic loss against whether or not the match is a control peak. We balanced the dataset to be 50% intron and 50% exon, sourcing sequences from the SpliceAI training dataset. As they were specifically trained to solve the eCLIP enrichment task, these AM-E models provide an upper bound on the performance one might expect any model to achieve on this task, though this bound is not necessarily tight as eCLIP-trained models may also learn technical eCLIP biases (e.g., related to the efficiency of crosslinking of different sequences) as well as true RBP-binding signal. When used as part of an end-to-end model of splicing, the AM-E models underperformed FM models using the same 18 motifs, achieving an accuracy of 50.3% vs 56.8% for the FMs, suggesting that, compared to the FM models, the AM-E models less accurately predict binding or less accurately predict binding that is relevant to splicing.

**Fig. 5:**
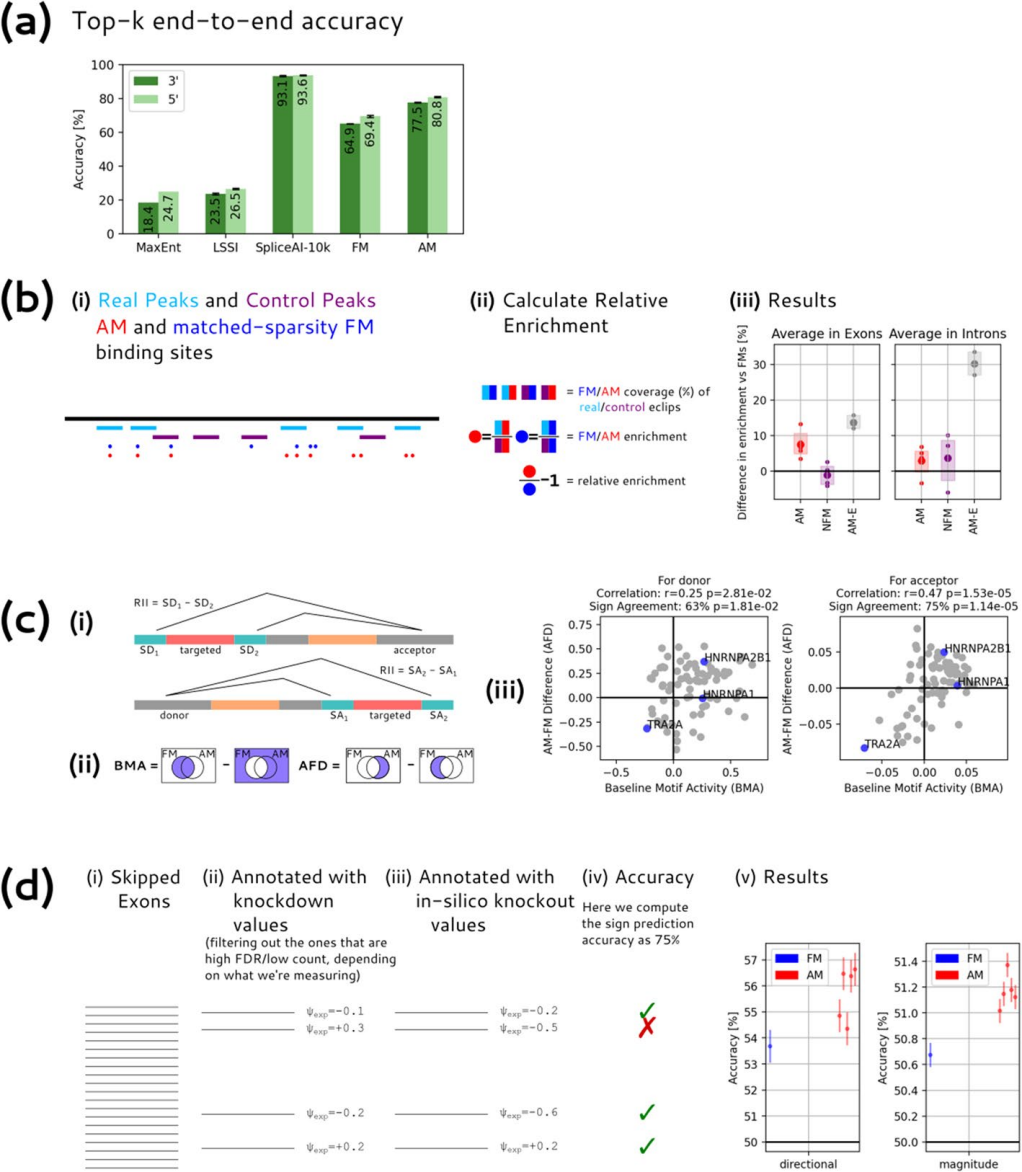
AM motifs perform better at predicting splicing, eCLIP peaks, MPRA activity, and knockdown data than FM motifs. The SAM model performs improves substantially at end-to-end prediction over existing RBNS-derived FM motifs, though it underperforms SpliceAI. In addition, the AM model, unlike SpliceAI, can also perform other motif-specific tasks, in all cases better than the FM motifs. **a** End-to-End Accuracy: Bars represent top-k accuracy of prediction of 3′SS (dark green) and 5′SS (light green) for different models trained on the SpliceAI training set and tested on the last 45% of the SpliceAI test set (the rest is used for validation. This 45% constitutes 37.14M nt across 730 genes). Error bars indicate the minimum and maximum performance among 5 replicates. The MaxEnt model is deterministic so does not have an error bar. **b** eCLIP peak prediction: (i) Schematic of a genomic region showing real eCLIP peaks (cyan) and randomly generated control peaks (purple). The control peaks are generated by shifting the locations of peaks for a given RBP to random positions in the same transcript. AM motifs (red) and sparsity-matched FM motifs on the sequence are also shown. (ii) We count peak overlap for each of the four comparisons depicted (in practice, we collect this separately per motif and average all 4 values over the set of motifs). We compute enrichments for each model, and then calculate the % increase from one to the other. (iii) Plotted are the mean relative enrichments across motifs, separately for exon and intron. AM-E models are provided as a reference for the maximum improvement we could hope to achieve using a motif model on eCLIP data. By this standard, the AM models achieve about 10% of the theoretical maximum performance improvement over FMs on introns and about 50% of the theoretical maximum performance improvement on exons. **c** MPRA activity prediction: (i) Schematic of the experimental setup from [36]. Reporters were used involving a pair of alternative 5′ (donor) splice sites, or a pair of alternative 3′ (acceptor) splice sites, such that the degenerate region in red is either included in the exon or spliced out (triangular lines depict possible introns). We ignore the other degenerate region (in orange). To compute the Relative Intron Inclusion (RII) score, we subtract the read count of the splice site that indicates the red region is in the intron from the one that indicates it is not. (ii) We compute the Baseline Motif Activity by looking at just FM sites and non-sites, as we are confident that FMs indicate binding. We compute the advantage gap for AMs by looking at the difference of uniquely AM and uniquely FM slices: note that this is a symmetric measurement. (iii) The relationship between these two metrics is significantly positive, on both the 3′ and 5′ data. We highlight the three well-established motifs from Fig. 6 in blue. **d** Knockdown modeling: (i) We use an existing dataset of skipped exons and corresponding in vivo knockdown results. (ii) Filtered for reliability both by looking at low FDR and by looking at high counts. (iii) We run our own model to compute in silico knockdowns. (iv) Our metric of interest is predictive accuracy of using the in silico values to predict the knockdown values, both using sign and magnitude above/below median. (iv) Results at predicting experimental sign and magnitude from in silico sign and magnitude. Different AM models indicate different replicates; error bars indicate 95% bootstrap confidence intervals

The results of these experiments (Fig. 5b) show that, across several seeds, our AM models improve on FMs in prediction of eCLIP binding, achieving about 50% of the theoretical possible improvement on exons and 10% on introns. These observations indicate that, in the process of modeling splicing end-to-end on the genome, the AM models have learned to better model the binding locations of RBPs in vivo. This observation supports that the constraints built into our model have encouraged modeling of biochemical features relevant to splicing.

### Splicing activity analysis

Next, we considered whether the AM models have captured more information relevant to splicing regulation in vivo relative to the FM models. For this purpose, we turned to data from a recent splicing massively parallel reporter assay (MPRA) experiment [36], using the randomized segments between alternative 5′SS motifs and between alternative 3′SS analyzed in this study (Fig. 5c). Since these regions are located in introns only when the distal splice point is used (SD1 or SA2), we can use the differences in splicing SD1–SD2 and SA2–SA1 as measures of much a sequence favors recognition as intron versus exon, referred to as the relative intronic inclusion (RII) of a given segment.

We consider a randomized segment to be bound by a certain RBP according to a motif model if the motif model predicts at least one binding site in the region. We did not use the Aggregator model in this experiment, since the sequences involved are far shorter than those handled by our model. Instead, we compute a baseline motif activity (BMA) for each motif by calculating the mean RII in segments that are RBP-bound according to the FM model minus the mean RII in segments not predicted to be bound. The BMA establishes an activity for an RBP as promoting recognition of bound regions as intron (if BMA > 0) or exon (if BMA < 0). To emphasize differences between the models, we compared sites bound only by the AM Model (AM\FM) and sites bound only by the FM model (FM\AM), with the difference in RII change referred to as the AM-FM Difference (AFD). Assuming that the BMA reflects the true activity of a factor, if ASA and BMA have the same sign, then the AM model better captures binding, while if they have the opposite sign, then the FM better captures binding.

We calculated AFD versus BMA in 5′SS and 3′SS datasets, averaged across five runs (Fig. 5c). For both categories, the correlation of the values and signs is positive and statistically significantly (*p* < 0.05 for 5′SS, *p* < 0.0001 for 3′SS) (the presence of some non-SRFs in the 79-RBP set may add some noise to the results). Thus, the AM models, in general, yield models that better predict splicing activity than FMs. This might result from better prediction of in vivo binding or better prediction of sites that have regulatory activity, for example. In a similar experiment using NFMs, BMA and AFD had the same sign 46% and 41% of the time on the 5′SS and 3′SS datasets, respectively, i.e., no advantage over the FMs. Therefore, these observations support that it is the training of the AMs in the end-to-end splicing training task that has improved their prediction of binding associated with splicing regulation.

### Analysis of RBP knockdown data

To further explore the relative ability of our models to infer the effects of RBPs on splicing, we used the RNA maps dataset from the ENCODE project [34]. This dataset consists of skipped exons annotated with change in percent spliced in (“delta *ψ*”) values with RNAi knockdown of a specific factor in human K562 and/or HepG2 cells; here, data from the two cell lines were combined to increase statistical power. Specifically, as described in Fig. 5d, we compute “in silico” delta *ψ* values by first predicting *ψ*, treating the model’s probabilistic prediction of splicing as a *ψ* value, and then subtracting from this value the *ψ* resulting from an “in silico knockout” where all occurrences of the relevant motif are zero-ed out (i.e., treated as absent) before passing the values to the Aggregator.

We use two separate targets in our experiment. First, we compute the proportion of exons for which the correct sign of delta *ψ* is predicted (Fig. 5d), focusing here on exons with FDR < 0.05, i.e., those which showed a significant difference in ψ following knockdown. Secondly, we compute the accuracy of predicting the magnitude of experimental delta ψ from the magnitude of in silico delta *ψ*, specifically predicting whether the magnitude is above or below the median of exons (filtering for experiments having at least 50 samples in both control and knockdown conditions).

This experiment has several limitations. First, our model has been trained entirely on “canonical” splice sites, which are mostly constitutive, and therefore may not capture unique features of alternative splicing. Second, our in silico knockouts are not a perfect match for the experimental system, in which the target protein level is depleted but not eliminated, and our model does not consider secondary (often compensatory) effects in which the expression of other SRFs changes in response to the knockdown. These considerations likely reduce the agreement between the in silico and experimental knockdowns. Despite this, we find that, in general, our models perform better than chance at predicting the sign and magnitude of delta *ψ* values and that AM models outperform FM models. These observations support that our model captures some aspects of the splicing regulatory activity of many RBPs and that this regulatory activity is better modeled with AMs than FMs.

### Interpretability and modularity

**Fig. 6:**
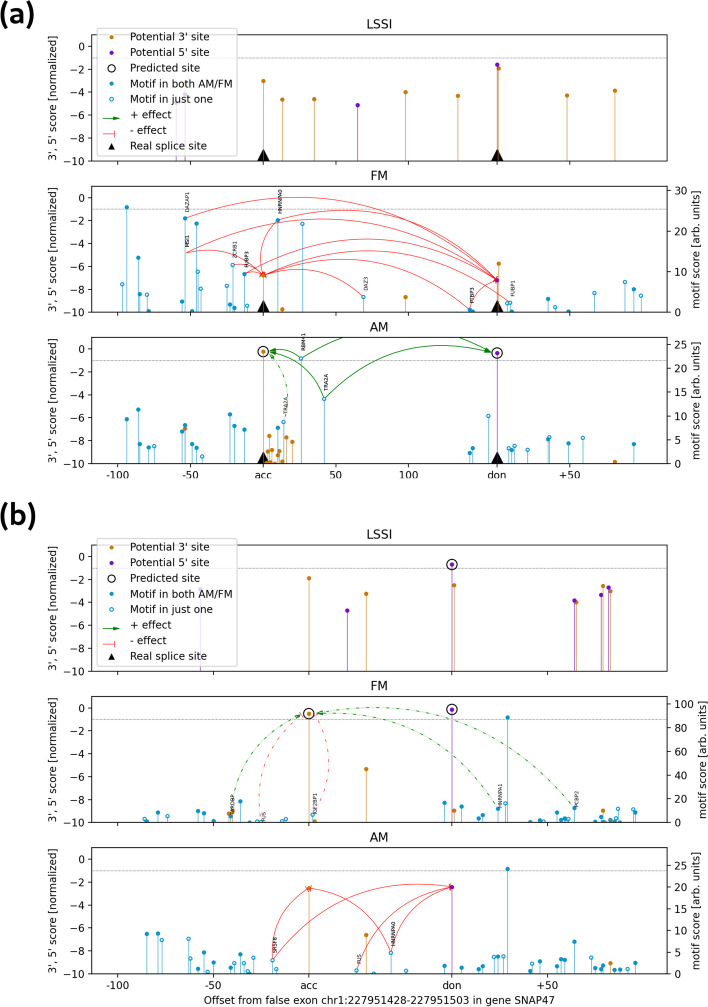
Examples of regulatory landscapes of FM and AM models for annotated and decoy exons. Predicted 3′SS and 5′SS are shown as orange and purple lollipops, respectively; height represents score, normalized so that − 1 is the top-k threshold for the model for each class; only splice sites scored above − 5 in the normalized score by at least one model are shown. Black triangles on the *x*-axis correspond to annotated splice sites, while circled predictions correspond to the predicted splice sites for each model. Motif predictions are shown as blue lollipops on the FM and AM plots, in relative affinity units. Filled circles represent motifs that are present in both models; empty circles are motifs that appear in only one. Solid arrows connect any motif to the corresponding splice site if an in silico knockout of the motif increases the splice site prediction probability by 1.5-fold or more (green) or decreases it by a factor of 2/3 or less (red arrows). At least the top 5 effects are shown for each exon, using dashed arrows to indicate those that do not meet the cutoff for solid line

To better understand the inner workings of our model, we conducted a series of computational experiments aimed at understanding the regulatory logic learned by SAM models. We first used in silico motif deletion to assess the regulatory impact of different motifs on each model’s predictions of individual genomic regions, comparing the FM and AM models with LSSI as a baseline reference; a sample exon and sample decoy exon are shown in Fig. 6a and Fig. 6b, respectively. For exon 54 of the *DNAH14* gene, the splice sites are too weak to be predicted by the LSSI model. However, motif deletions showed that the AM model predicts both splice sites correctly primarily because of positive impacts from RBM41 and TRA2A motifs located inside the exon. The FM model identifies some of the same motifs (e.g., the RBM41 motif but not the TRA2A motif) and considers additional negative-regulatory motifs, causing it to miss the exon.

In the second example, a “decoy” (false) exon is predicted by the FM model in the gene *SNAP47*, with the 5′SS also predicted by the LSSI model, but the AM model correctly does not predict the decoy exon because of inhibitory effects of three RBP motifs (associated with SRSF8, FUS and HNRNPA0) not identified by the FM model. These examples were curated for compactness and to illustrate cases where the AM and FM models differ, but such a “putative regulatory landscape” can be generated for any exon or genomic region of interest, with uncertainty estimates possible using several different model seeds. Additional examples are shown in Additional file 1.

**Fig. 7:**
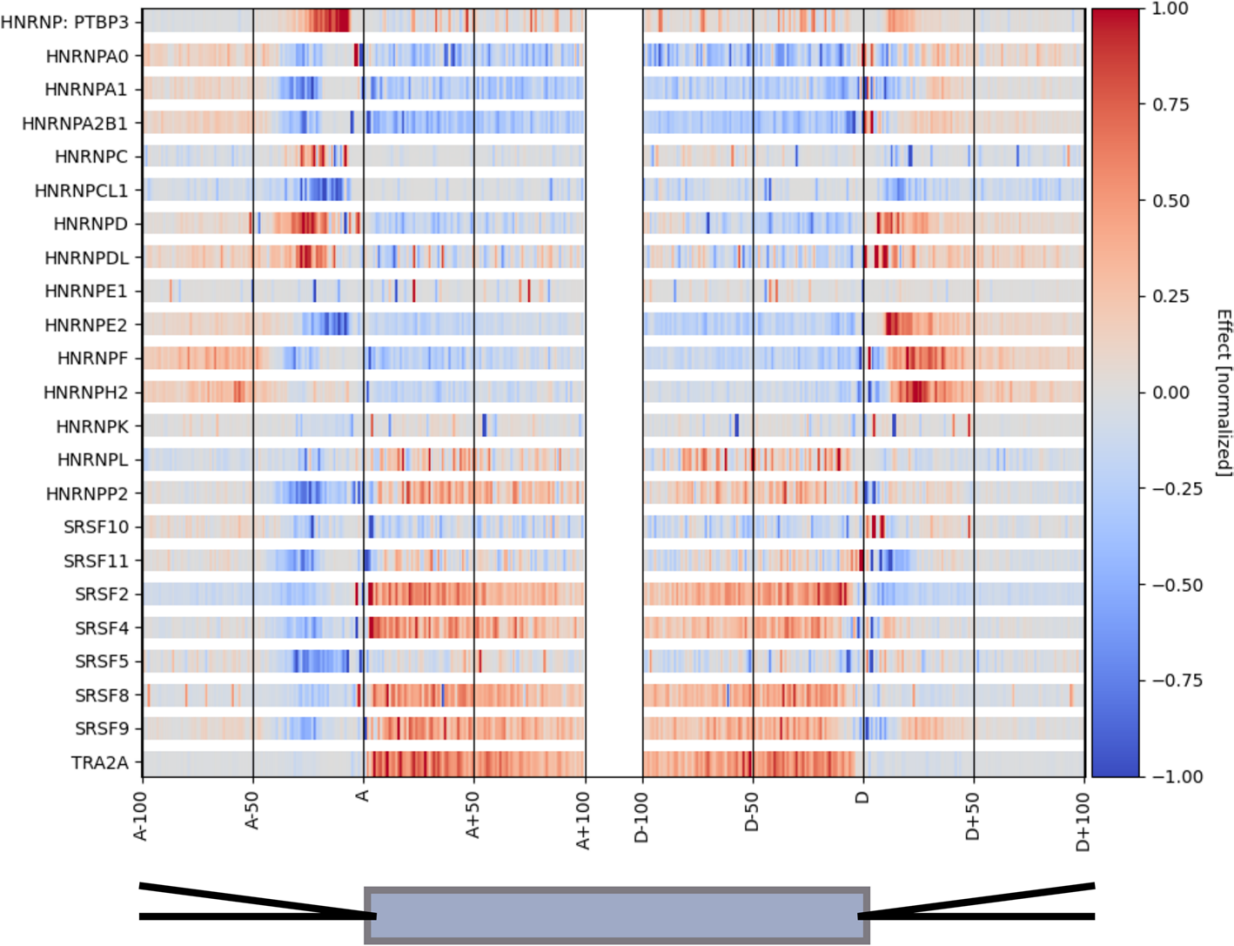
Inferred RNA maps for selected RBPs. To create this plot, we performed in silico knockdowns on the binarized version of our model and measured changes to splicing. We then aggregated across several positions to create overall effects at each position in the meta-exon. The results here are normalized for each motif individually, with an absolute value of 1 corresponding to the 99th percentile across positions. This normalization enables us to see the RNA map for each motif. The *x*-axis here is in two parts, with the left half being relative to 3′ sites (A) and the right half being relative to 5′ sites (D); a schematic exon is shown at bottom

We also analyzed the aggregate effects of individual motifs on splicing (Fig. 7). For purposes of comparison, we show the motif effects predicted by our model for all hnRNP, SR, and SR-related proteins studied. These protein classes were chosen as they have well-understood effects on constitutive splicing and cassette alternative exons, with SR and SR-related proteins generally having positive effects and hnRNP proteins having negative effects on exon inclusion when bound in exons [1, 37, 38]. Overall, the analysis of motif effects in our model matched these expectations well. For example, 5 of the 8 SR and SR-related proteins studied showed strong positive activity (dark red) in exons (SRSF-2, SRSF-4, SRSF-8, SRSF-9, and TRA2A), while 2 had weaker net positive activity (SRSF-5 and SRSF-11), and one had negative activity (SRSF10) (Fig. 7). Thus, the expected direction was observed in 7 out of 8 cases, and even the 8th case appears to be correct, as SRSF10 is an atypical SR protein that can repress splicing in exons [39]. For hnRNP proteins, we observed broad agreement with the expectation of negative activity, with 9 of 15 proteins showing negative activity in exons (mostly blue), 3 showing positive activity (PTBP3, HNRNPL, and HNRNPP2), and the remaining 3 showing a mixed pattern (HNRNP-C, HNRNP-DL, and -E1). Thus, of 23 SR/SR-related and hnRNP proteins studied, 16 (or 17, counting SRSF10) exhibited patterns of activity consistent with expectations for their class. Similar results obtained for FM models (Additional file 1: Figure S5), indicating that our modular architecture captures the splicing activity of RBPs.

A direct side-by-side comparison with the RNA maps based on eCLIP and knockdown data from ENCODE [34] yielded fairly strong overall concordance (Additional file 1: Figure S6). The small number of discrepant cases might result from technical biases in eCLIP or RBNS, or from “aliasing” issues, where a motif associated with the RBP that was CLIPped and knocked down is also bound by another protein with distinct splicing regulatory activity.

### Prediction of other classes of splice sites

**Fig. 8:**
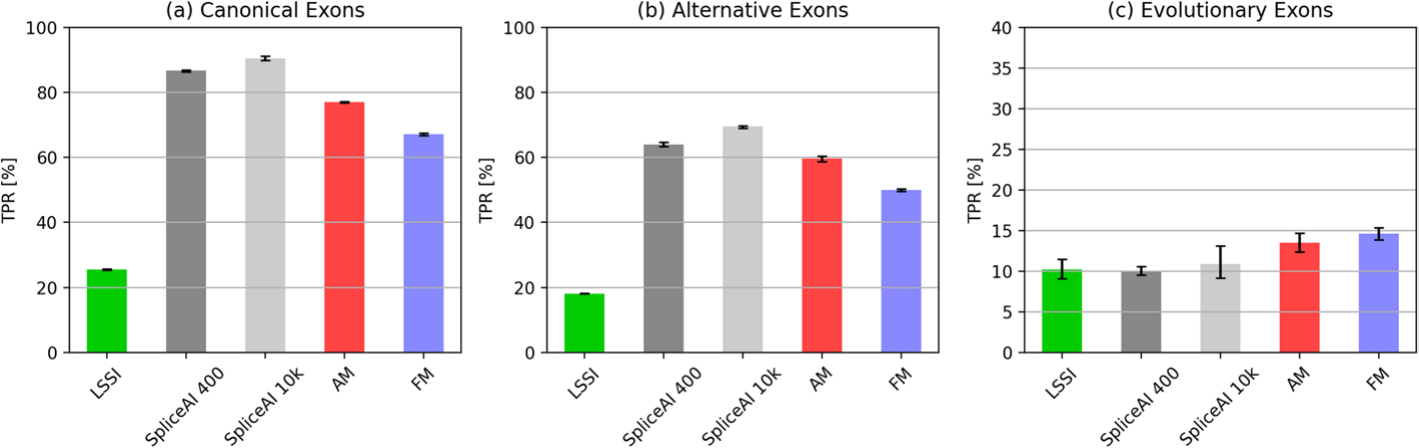
Accuracy for different classes of exons. Top-k accuracy of splice site prediction for models trained on canonical splice sites applied to a canonical exons, b alternatively spliced exons: and c evolutionarily new exons (note that the *y*-axis range was reduced for this panel to facilitate comparison). Here, the value of “k” used for top-k accuracy is the union of the numbers of splice sites across all 3 classes, rather than per splice site type: this approach ensures a consistent threshold across the three conditions. Error bars are 95% bootstrap intervals of the mean across 5 replicates

The transcript annotations used in the majority of our model training and testing were taken from the SpliceAI “canonical” gene annotations, which include a single annotation for each gene based on the principal transcript from that gene and therefore include predominantly constitutive exons. But most human genes contain additional alternative exons, and some contain evolutionarily new exons, both of which are likely to be more challenging to predict. Previously, it was reported that SpliceAI yields somewhat weaker predictions on alternative exons relative to constitutive exons [18], and we observed a systematic shift toward lower accuracy for all models on alternative exons. While further study is needed, likely contributors to this pattern are that alternative exons have weaker splice sites and a regulatory element composition that is less biased toward positive-acting elements than constitutive exons (Fig. 8) [40].

We also analyzed a previously described set of evolutionarily new internal human exons (i.e., genomic segments spliced as exons in humans but not spliced as exons in rodents or other mammals), which we anticipated would be difficult to predict due to their weaker splice sites, frequent alternative splicing, and sequence features that are reminiscent of their intronic origins [41] (the splice sites of alternative exons and evolutionarily new exons overlapped to only a small extent with the canonical splice site dataset (Additional file 1: Figure S7)). As expected, the splice sites surrounding evolutionarily novel exons proved far more challenging to predict for all models (Fig. 8c). On this challenging task, our interpretable models (AM, FM) performed moderately better than others.

## Conclusions

Here, we have shown that a neural model using core types of RNA elements known to contribute to splicing mechanisms can achieve fairly high accuracy and can learn binding and regulatory activities of RBPs. Indeed, we find that the AM models actually provide improved descriptions of the in vivo binding and regulatory activity of RBPs involved in splicing. Furthermore, our approach can infer RNA maps for splicing factors purely from in vitro binding data and the genome sequence, i.e., without requiring the CLIP and knockdown/RNA-seq data typically required for RNA map inference. We can also generate a putative regulatory landscape for any exon, which could have applications to genetic variant interpretation or design of therapeutic antisense oligonucleotides to perturb splicing. Furthermore, the general AM approach could potentially be applied outside of splicing to study other RNA- or DNA-binding proteins for which a defined regulatory activity and suitable training set are available.

## Methods

### LSSI architecture

We structure our LSSI models as simple feedforward neural networks that first map their input to 100 hidden neurons, then have 5 hidden 100-wide layers with ReLU activations, and then finally mapping to a prediction output. These models are trained on the same training dataset as our other models, using 10 epochs, a batch size of 150, and a learning rate of 10^-3^.

### Derivation of entropy bound

Here, we provide the derivation of the bound *H/L* ≤ *M[H(B(δ)) + δ η]*, where *B(x)* represents the Bernoulli distribution with probability *x*, *δ*_*i*_ represents the sparsity of motif *i*, *η*_*i*_ represents the entropy per nonzero value of channel *i*, and *η* represents the entropy in the overall distribution of activations across channels:

$${\displaystyle \begin{array}{rlr}H\left(\textrm{mo}\textrm{tifs}\right)/L &\!\!\!\le {\sum}_iH\left(\textrm{mo}{\textrm{t}}_i\right)/L& \left[\textrm{independence}\right]\\ &\!\!\!\le {\sum}_iH\left(B\left({\delta}_i\right)\right)+{\delta}_i{\eta}_i+\left(1-{\delta}_i\right)0& \left[\textrm{maximal}\ \textrm{usage}\ \textrm{of}\ \textrm{channel}\right]\\ &\!\!\!=\left({\sum}_iH\left(B\left({\delta}_i\right)\right)\right)+M{\delta}_{\eta }& \left[\textrm{by}\ \textrm{definition}\ \textrm{of}\ \eta \right]\\ &\!\!\!=M\left(\frac{1}{M}{\sum}_iH\left(B\left({\delta}_i\right)\right)\right)+M{\delta}_{\eta }& \\ &\!\!\!=M\ {E}_{x\sim \left\{{\delta}_{1\cdots }{\delta}_M\right\}}\left[{\sum}_iH\left(B(x)\right)\right]+M{\delta}_{\eta }& \\ &\!\!\!\le MH\left(B\left(\delta \right)\right)+M{\delta}_{\eta }& \left[{\textrm{Jensen}}^{\prime}\textrm{s}\right]\end{array}}$$

### Adjusted motif model

We compute the binding sites in the adjusted motif (AM) model by adjusting the scores for already relatively high-scoring FM binding sites, allowing *k* times as many FM sites as we will eventually use, allowing our AM model to filter out a subset of binding sites among plausible sites provided by the FM model, as follows:

$${\displaystyle \begin{aligned}f&= FM(x) \\ {}{f}^{\prime }&={\text{Sparse}}\left(f, k\delta \right)& \\ {}a&= Adj(x)& \\ {}{y}&={f}^{\prime}\bigoplus a& \left[x\bigoplus {y}=1\left(x\ne 0\right)\left(x+{y}\right)\right]\end{aligned}}$$

### Neural models trained on RBNS data

Given that the FMs are derived via the RBPamp algorithm from the RBNS dataset, we also tested the hypothesis that a neural version of motifs trained from the RBNS dataset could better represent protein binding. These neural motifs are also used as baselines to compare the performance on RBNS binding from AMs. We formulated the training task of the RBNS dataset as a binary classification task. Given a sequence from the RBNS dataset, we measure the accuracy of classification of the sequence being from the protein-bound or input RNA pool in the original RBNS experiment. Specifically, we select the sequence binding dataset for each protein with the highest 5-mer enrichment (“R value”) from the RBNS dataset. We trained our “Adjusted Motif” architecture on this dataset but did not enforce sparsity. This becomes effectively just the sum of a PSAM signal and a learned neural model. This approach was used because it allows the learned model to have the advantage the AMs have of being able to take advantage of the PSAMs, but we do not want to constrain the model to be similar to PSAMs since it is being trained on the RBNS data directly.

We also separately trained 4-layer convolutional neural networks with two sliding window width choices, width = 11 (NM11) and width = 14 (NM14), in order to ensure that we are not too heavily tied to a single model architecture. Testing these models with a sparsity of 0.18% calibrated on the genome, we find that the NM14 and AM models improve on the RBNS dataset: gaining an improvement of 0.33% and 1.76% respectively, while the NM11 does not (it yielded a −0.02% change in performance). Our NFM models also improved on eCLIP, though the results were highly inconsistent. On average, they achieved about 6% of the improvement of the AM-E models, but their overall range of improvements across 4 replicates overlaps that of the AM.

### Analysis of MPRA data

In the case of the 5′SS MPRA data, the leftmost 3 bases in the targeted region do not have 10 bases of context to their left (as there are only 7 bases in the SD1 region). Since our AM models are 21-wide, they require 10 bases on either side of any site where we would predict a motif, we removed the leftmost 3 positions from consideration. For consistency, we did this for both the AMs and the FMs (it would not be strictly necessary for FMs, since they are only 11-wide).

### Analysis of exon classes

We consider both alternative and evolutionarily new exons whose splice sites were both located inside their respective gene boundaries as determined by the canonical dataset. If an exon’s associated gene was not included in the canonical dataset, it was also excluded from consideration. The majority of the resulting splice sites were not included in the canonical dataset, though we found 76 splice sites in common between the canonical SS and the evolutionarily new SS and around 180 SS in common between the alternative SS and the evolutionarily new SS, as shown in Fig. 7.

### Supplementary Information

**Additional file 1.** Supplementary text and figures.

**Additional file 2.** Review history.

## Data Availability

The computer code needed to reproduce the analyses shown in this manuscript is available on GitHub at https://github.com/kavigupta/sam/tree/main/spliceai/Canonical [42]. The code is licensed under GPL 3 and has Zenodo ID 10.5281/zenodo.10393043 [43]. We used the following data sources in this work: the HG19 human genome sequence [44], SpliceAI “canonical” transcript for training and evaluation and models for comparison [18], Maximum Entropy models for comparison [5], the RBNS binding data for training and evaluating models and the RBD count data for analyzing width [26], eCLIP and knockdown data for evaluation of our models and RNA Maps for comparison [34], MPRA data for evaluation of our models [36], alternative exons data from GTEx to evaluate our models [45], evolutionary exons to evaluate our models [41], and RBNS PSAMs [27] and RNACompete PSAMs [46] to use as FMs.
