# Supplementary material for: Improved modeling of RNA-binding protein motifs in an interpretable neural model of RNA splicing
Source: Genome Biol. 2024 Jan 16;25:23. doi: 10.1186/s13059-023-03162-x (PMC10790492; doi:10.1186/s13059-023-03162-x)
Supplement: Supplementary file 1 — Additional file 1. Supplementary text and figures. [file 13059_2023_3162_MOESM1_ESM.pdf]

Supplementary Text and Figures

Entropy of nonzero motif values

To bound the number of bits of entropy in the nonzero motif binding sites, we run a binning experiment where we bin the nonzero motif binding sites into a certain number of bins based on rounding to the nearest k, then use the mean of each bin as the value for all elements of the bin. We then calculate both the empirical entropy of this distribution as well as the drop in accuracy when this rounding scheme is employed (Figure S1). In general, we find that FMs have no entropy, whereas AMs have similar empirical entropies, both about 1.5-2b/activation. Both are below the 2b threshold that is needed for our 0.18% sparsity to translate into a sub-1.91b/nt entropy.

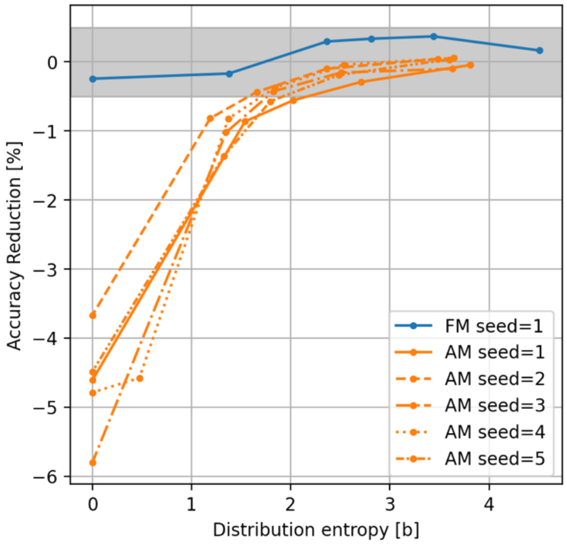

Figure S1. The effects of score binning on FM and AM model predictions. On the x-axis is the number of bits of entropy in the distribution, on the y axis is the reduction in accuracy from the original. We consider a reduction or increase of 0.5% to be within the noise region, as modifying a model in an open-loop format in any way will lead to some degradation in performance.

Effect of Aggregator Architecture

While the focus of our paper is on the Motif model and sparsity bottleneck rather than the Aggregator, we feel that our Aggregator architecture (referred to here as attention + recurrence, or A+R) has some benefits in terms of encouraging the motif models to behave more like RBP

binding predictors. We use the SpliceAI-10k model as an aggregator as a baseline in order to demonstrate these properties. The SpliceAI model is a very flexible convolutional model, and also has a slightly longer context window which allows it to perform somewhat better than our Aggregator on end-to-end splicing. However, our primary focus was on motif quality. To explore this question, we performed a module substitution experiment (Supplementary Figure S2). In this experiment, we extracted motif models from AMs trained with either the A+R or the SpliceAI model as aggregator, and then we paired these with either the A+R or SpliceAI aggregator trained on FMs. What we find is that regardless of the ultimate aggregator used, the Motif Models trained with the A+R aggregator outperform those trained with the SpliceAI aggregator. This observation indicates that our A+R aggregator improves overall motif quality and thus interpretability, though it's end-to-end accuracy does not match that of SpliceAI.

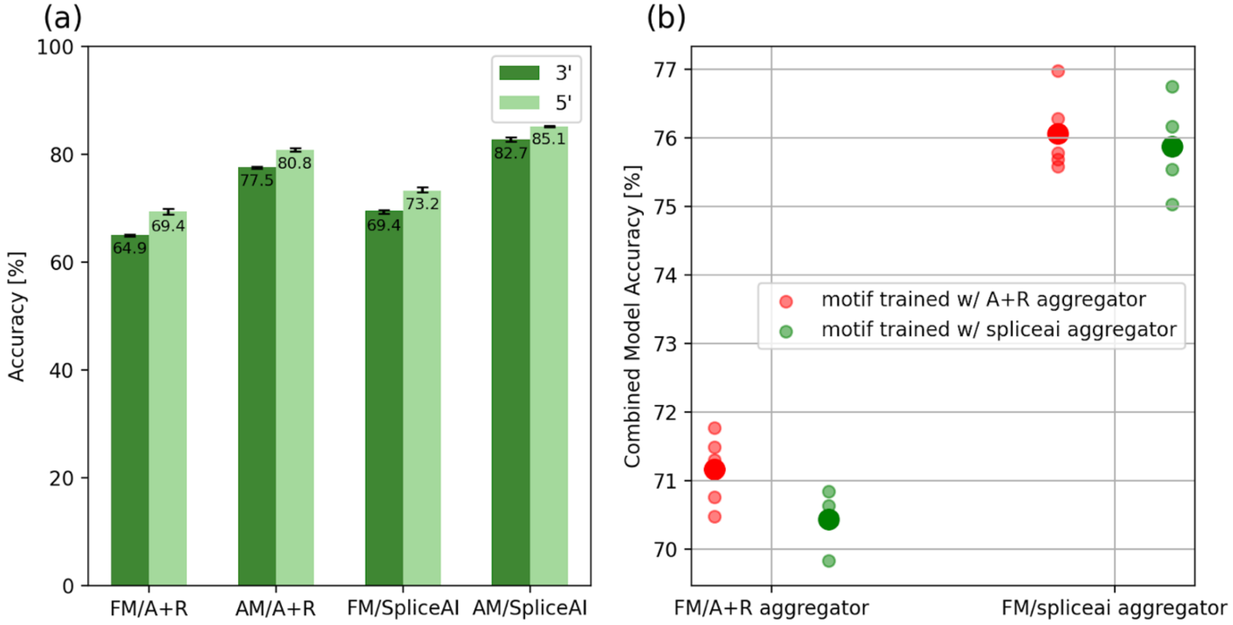

Figure S2. Effects of substitution of Aggregator architectures on performance.
(a) End-to-end splicing results for FMs and AMs using both the A+R and SpliceAI aggregator.
(b) Module Substitution Experiment results for combining A+R and SpliceAI-trained AM motif models with FM aggregators of both varieties.

Motif Width

Our Adjusted Motif Models take in a context length of 21 nt. We find that this length – somewhat longer than typically used to model RBP binding – yields a pronounced improvement in performance. In this section, we contrast 21 nt models with 13 nt ones, length that more closely

approximates the 11 nt used in the RBPamp PSAMs (Jens 2022). We note that shrinking our motifs from 21 to 13 reduces accuracy by ~1.7%. Below, we analyze several possibilities for what might case this difference.

Motif architecture. First, we examined the motif models themselves. The shorter AM is composed of a smaller number of Residual Units, and as such has a theoretically smaller computational capacity. However as seen in part (a) of Figure 3a, adding in additional layers that do not extend the width of this model does not improve performance, and in fact slightly reduces it.

Secondary structure. Another hypothesis is that RNA secondary structure can be more easily captured by longer motifs. To test this hypothesis, we provided the mean basepairing probability to the algorithm from a standard thermodynamic RNA secondary structure prediction algorithm. To do so we began with sequences of length 40 nt, adding 30 nt of context to either side, then used RNAFold (Lorenz 2011) on the 100 nt sequences, computing the probabilities of all base pairs in secondary structure. We then sum across to find the probability of any given base being bound in secondary structure, and discard the 30 nt on each side, leaving the more reliable center 40 nt. We find that this improves performance by about 0.4%, but this could provide some information about the sequence so is difficult to interpret. To properly control for this, we also provide an alternate source of information about the sequence, where we run the exact same procedure, but first swap all A and C bases in the sequence. This produces a similar kind of signal from an informational perspective, but is not in any way related to secondary structure. We find that there is no difference in the performance of this model and the model using the original secondary structure. This experiment suggests that capturing the overall secondary structure potential of a region is not particularly helpful in prediction and is therefore not likely to explain the advantage of wider motifs, though it doesn’t rule out that some other aspect of RNA secondary structure might be involved.

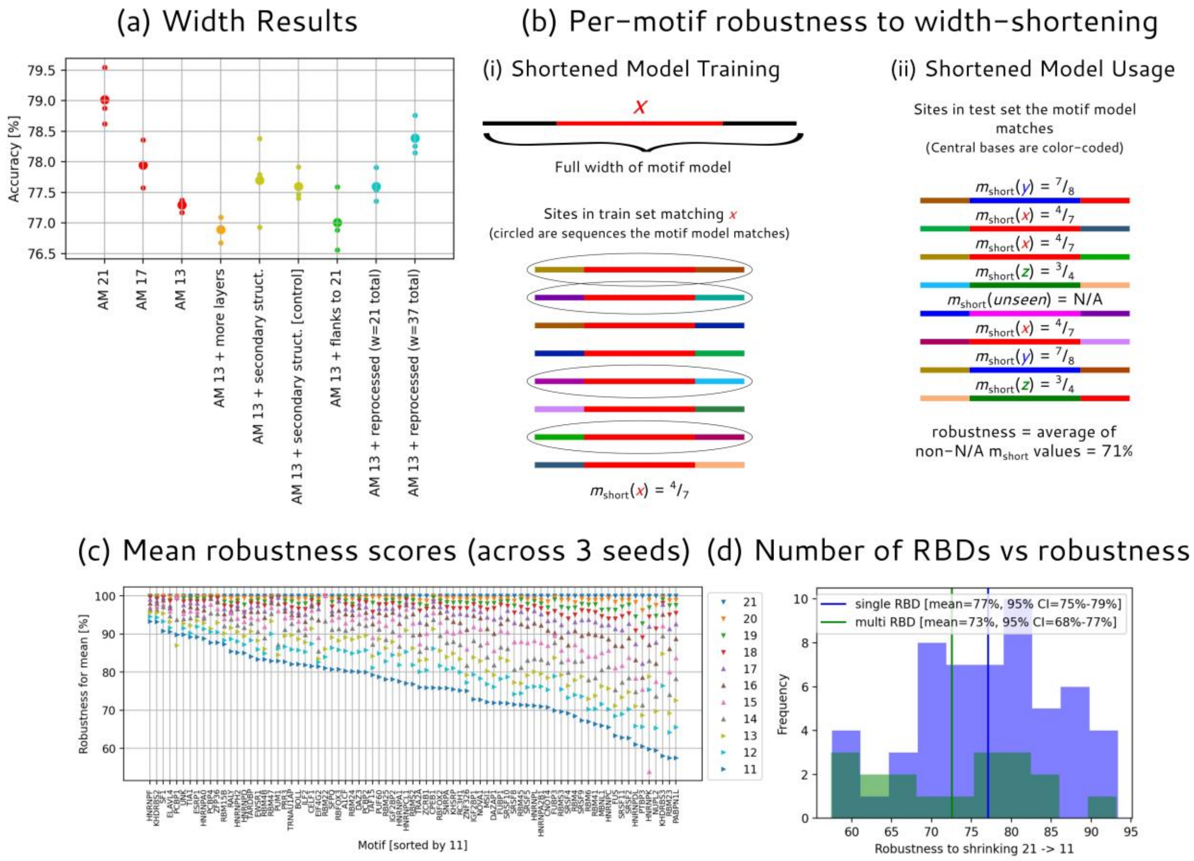

Figure S3. Motifs of width 21 are optimal overall.

(a) Accuracy for several different motif models. In general, we find that greater width leads to greater accuracy, and this cannot be made up with more layers, secondary structure, a flank-included motif model, or a reprocessor layer. (b) (i) Shortened motif model training: for all core sequences of length w – a – b, we scan the training dataset (from the genome) and find all instances of matches, then find how many of them are at the center of matches of the full motif model. Our shortened model outputs this fraction. (ii) Testing: we then average the values of the shortened motif model on all matches of the motif model. (c) Motif width-reduction robustness values for a variety of widths, from 21 down to 11. Below 11 the numbers tend to drop far more quickly as we start clipping the FMs. The motifs here are ordered by their robustness at 11, and the values here are taken as a mean over several other motifs. (d) Histogram of single and multi-RBD motifs' robustness to shrinkage. There is no apparent relationship between robustness to shrinkage and number of RBDs.

Flanking sequence context. We also considered the possibility that there is some broad flanking context that is useful in predicting whether an RBP will bind a site. For example, perhaps an RBP binds more frequently in AT-rich sequences over GC-rich sequences (Dominguez 2018). To explore this possibility, we trained a model that uses the core 13 nt AM, but adds in a 21-wide "flanking model", which is effectively a PSAM but with the restriction that the ratios between the log affinities of each base are fixed at each position – in effect, requiring that the same preference be applied to each position, though with potentially different magnitudes. This approach also failed to increase accuracy, suggesting that increased width being more accurate is not closely related to general flanking base composition.

Cooperative/competitive motifs. Finally, we investigate the possibility that our longer motif models are picking up on competition between motifs – effectively, that the "21 wide motifs" actually represent a smaller motif that has learned to recognize other motifs in the same window. We investigate this using a Reprocessed Motif model, which takes the outputs of the 13-wide AM model and runs them through another convolutional network. When we make this network 9 wide, in order to make our overall width 21, we end up with slightly better performance (+0.3%), but less than that achieved by a 17-wide motif. Even when we make the Reprocessor layer 25 wide (total width of 37), we are unable to improve to the same performance as a 21-wide network. This result argues against the idea that other motifs are being picked up in the 21-wide window, but has the caveat that motifs outside our considered set might be learned.

Robustness Scores

We also investigate the width per-motif by using our robustness to width-shortening method. We compute this for any number of bases removed from the left and the right by "training" a shortened motif model as depicted in Supplementary Figure S3b. In doing so, we compute for every shortened sequence the probability that it is part of a true motif binding site. We then "evaluate" this shortened motif model by computing the mean score on all true binding sites of the motif model on the test set. We then compute the true robustness metric at a certain length by taking the maximum over all available shortened windows. We plot a graph of this metric, averaged across three separate AM models, for all motifs and various widths. Using this metric, we can test another hypothesis, that our adjustment model is using a larger width in order to pick up on multi-RBD motifs. However, if this were the case, we would expect much lower

robustness to shrinking among models with multiple RBDs, which was not observed (Supplementary Figure S3d).

Taken together, these experiments failed to yield a compelling explanation for the performance difference between 21 and 13 wide models. It is possible RBP motifs have longer binding sequences than previously thought, or some other factor we have not considered could be underlying this phenomenon.

eCLIP metric

To compute intron/exon-controlled eCLIP enrichment for a set of predicted motif binding sites, we performed the following procedure. First, we created a “control peaks set” that consisted of the same number of peaks per motif, but distributed at random throughout the test set. We then split up both the real and control peak sets into intronic and exonic sets based on whether they were entirely in the intron or exon (peaks that crossed a splice boundary were discarded). Next, we computed the fraction of eCLIPs under each of the 2 x 2 x 18 conditions (real/control, intron/exon, motif) that were covered by the given motif binding sites, where coverage was defined as containing a nonzero value. We then averaged across motifs to leave us with 2 x 2 values, the $\text{coverage}_{\text{real},x}$ and $\text{coverage}_{\text{control},x}$ for x in [intron, exon]. The final enrichment values produced are $\text{enirchment}_{x} = \text{coverage}_{\text{real},x} / \text{coverage}_{\text{control},x}$.

The need for splitting up introns and exons comes from the well-known bias of eCLIP towards exonic peaks (Uhl et al., 2020), which might otherwise bias our results if, for example, the AMs were promoting exonic binding sites over intronic ones. Averaging across motifs was done to reduce the variance of the experiment. Finally, the control eCLIP set was done to ensure that we had an interpretable final result, enrichment, rather than coverage. This, however, did not make our metric insensitive to the sparsity of the motif binding sites; as less sparse motif binding sites would often get lower enrichment values as there was less room for them to provide signal.

As a result, we made sure to match the sparsity of the FMs exactly on each motif before taking the relative enrichment under intron and exon, which was computed as $\text{relative\_enrichment}_{x} = (\text{enrichment}_{\text{tested model},x} – \text{enrichment}_{\text{FM},x})/\text{enrichment}_{\text{FM},x}$. Attempting to somehow take a weighted average of these conditions was problematic as introns are far larger but exons are generally closer to splice sites and thus each nucleotide is on average more important in splicing. Thus, we opted instead to present them separately.

Non-interpretability of SpliceAI model

Initially, we attempted to extract a directly interpretable model from SpliceAI. However, we found that considering sequence perturbations was not clarifying, as it appears that the model uses information widely distributed across the sequence. To demonstrate this, we performed the following experiment, using SpliceAI-400 for ease of calculation. In the experiment, we considered strong true positive splice sites: those which SpliceAI predicts correctly with at least 10% margin over the top-k threshold. For each given splice site, we computed a “perturbation swing” at every input base, defined as the maximum absolute difference in the predicted probability of the splice site that can result from substituting the given base, testing each of the three alternate bases. We then varied a threshold on the perturbation swing and randomized all bases whose swing values were below this threshold, in essence randomizing the portions of the sequence that are considered less important by SpliceAI.

The results of this experiment, averaged across several different splice sites, are depicted in Supplementary Figure S4a. At a swing threshold of even as low as 1%, we find that 60% of the sequence falls below this threshold, and that randomizing these bases leads to a 25% decrease in accuracy. Since we selected for robust splice sites with probability at least 10% above the threshold, this indicates that many positions are being used in tandem to produce the overall signal for splicing. Thus, single perturbations are insufficient to explain SpliceAI predictions in general, even for SpliceAI-400.

Additionally, we considered whether single base perturbations are sufficient to infer motif locations, potentially obviating the need for an explicit motif model. To test this idea, we determined the distance from the nearest motif (using the FM model with density 0.18% to define the motif positions; this is effectively the RBNS motifs) and in each distance category we computed the mean perturbation swing. We excluded the 40 bases closest to the splice site of interest to ensure that the core splice motif is not contaminating the results. Surprisingly, we found that distance from the nearest motif center positively rather than negatively correlated with perturbation swing, as seen in Supplementary Figure S4(b)(i). This was true even controlling for LSSI sites (splice site motifs scored with log probability -10 or higher by the LSSI model), by looking only at positions that are at least 20 nt away from the nearest LSSI site. We can conclude that while SpliceAI presumably uses information about SREs to make splicing predictions, it clearly is using other information to a greater degree, leading to its predictions being uninterpretable from the point of view of the SREs considered here, which include motifs for many canonical splicing factors of the hnRNP, SR and SR-related protein classes likely representing a substantial portion of splicing regulatory motifs. As a check of our metric, we

show in Supplementary Figure S4 (b)(ii-iii) that this property is unique to SpliceAI and does not apply to either of our models, both of which show the opposite trend, starting with similar absolute swing magnitudes, with perturbation swing declining rather than increasing as we move further from the nearest motif.

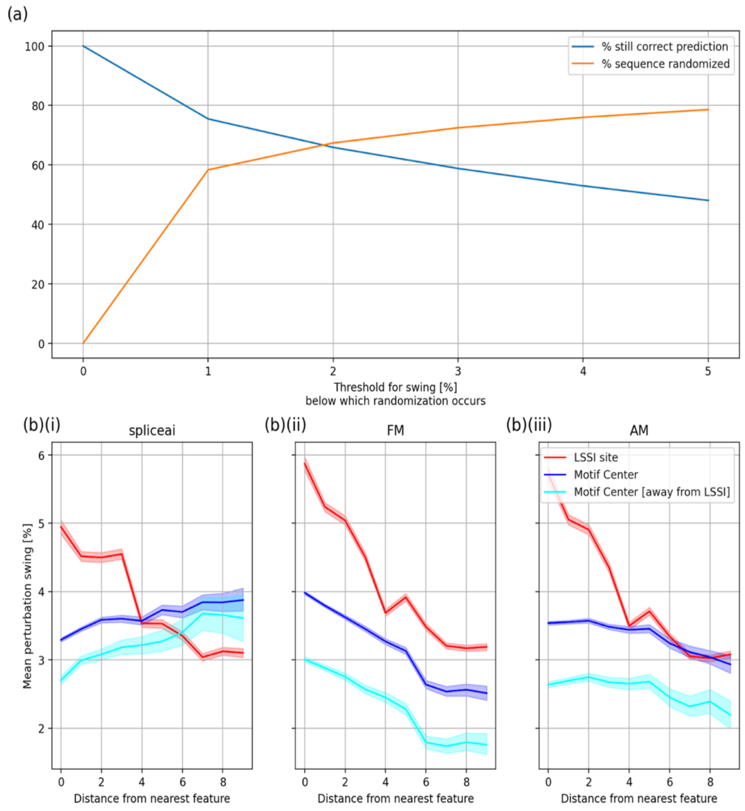

Figure S4. Non-interpretability of SpliceAI. (a) Accuracy of SpliceAI-400 applied to partially randomized sequences, as described in text. (b) Mean perturbation in score resulting from in silico mutations at different distances from LSSI sites (splice site motifs, red), RBP motif centers (dark blue), or RBP motif centers located at least 40 nt from LSSI sites (light blue) The distance from the nearest feature is shown on the x-axis (in nt), and the mean perturbation swing on the y-axis.

Use of RNACompete PSAMs

To ensure that our results are not specific to RBNS PSAMs, we also consider RNACompete and a hybrid model where we merge the RNACompete and RBNS sets, preferring RBNS motifs when we have both available. We sourced RNACompete PSAMs from (Ray 2013), filtering for vertebrate RBPs. We consider the same RBP from different species to be different motifs. As such, we end up with a set of 97 motifs in the RNACompete set and 143 in the hybrid set. We

calibrate our sparsity to have an approximately equivalent entropy as 79 motifs at 0.18% sparsity, and achieve an AM accuracy of 78.7% on RNACompete and 79.0% on our hybrid set. This demonstrates that our method is not specific to the RBNS motifs and works across different motif sets, but since these are larger motif sets and do not produce a change in performance, we continue to work with the smaller 79 motif RBNS set.

Additional examples of cases where AMs and FMs differ in prediction

Examples are provided in the Additional file "Additional Examples.zip". Several examples of situations where AMs predicted an exon correctly and FMs did not. Exons are selected for size, LSSI incorrectness, FM incorrectness, AM correctness, the presence of TRA2A in the exon (for positive examples; we did so because this motif has a well-understood and strong effect so it serves as a sanity check for the correctness of the splicing mechanism) and being on the positive strand (for convenience when viewing on a genome browser).

Supplementary Reference

Lorenz, R., Bernhart, S.H., Honer Zu Siederdissen, C., Tafer, H., Flamm, C., Stadler, P.F., and Hofacker, I.L. (2011). ViennaRNA Package 2.0. Algorithms Mol Biol 6, 26. 10.1186/1748-7188-6-26.

Uhl, M., Tran, V.D. & Backofen, R. Improving CLIP-seq data analysis by incorporating transcript information. *BMC Genomics* **21**, 894 (2020). https://doi.org/10.1186/s12864-020-07297-0.

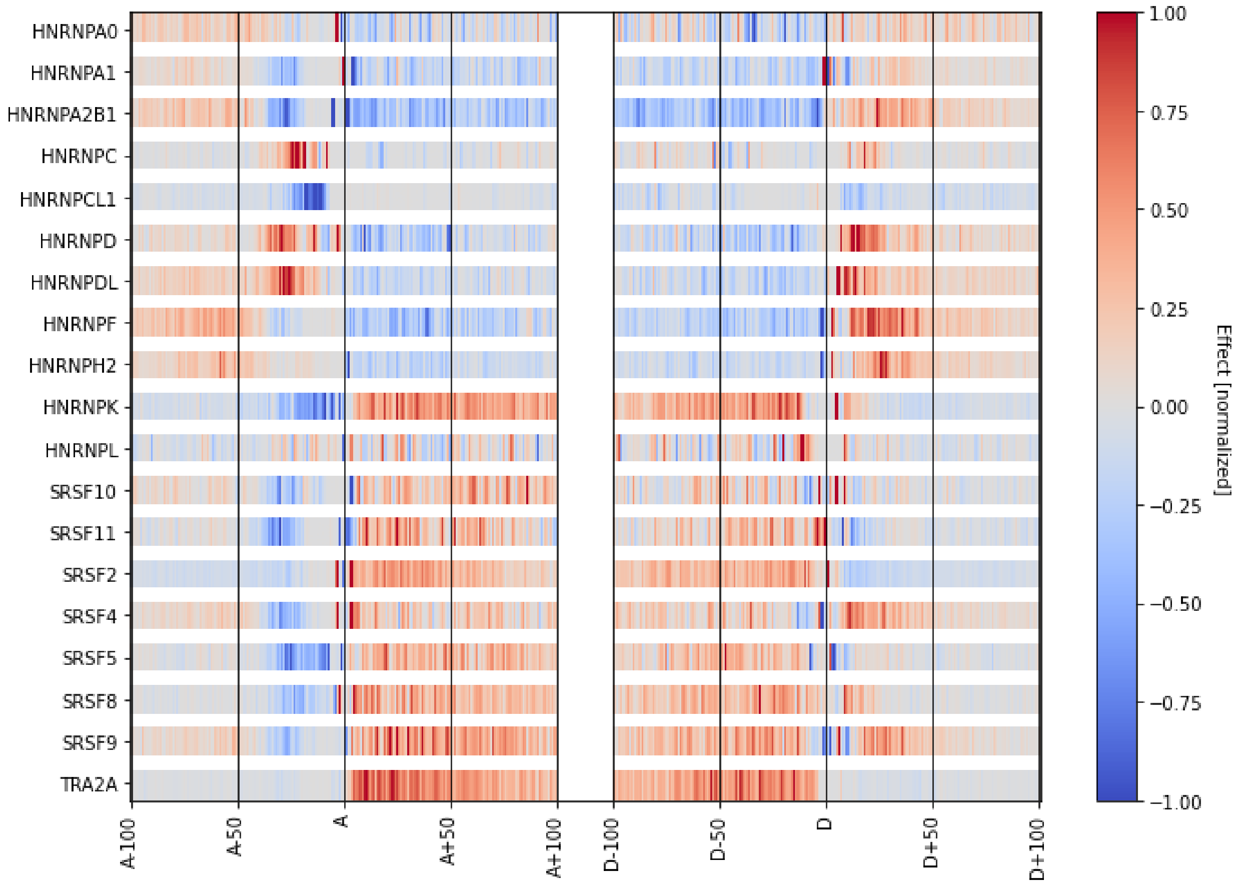

Figure S5. RNA maps using FM models. Similar to Figure 7. Data averaged over 5 FM replicates. Like AMs, the effect is broadly consistent with literature, with potential exceptions for HNRNPCL1, HNRNPK, and HNRNPL.

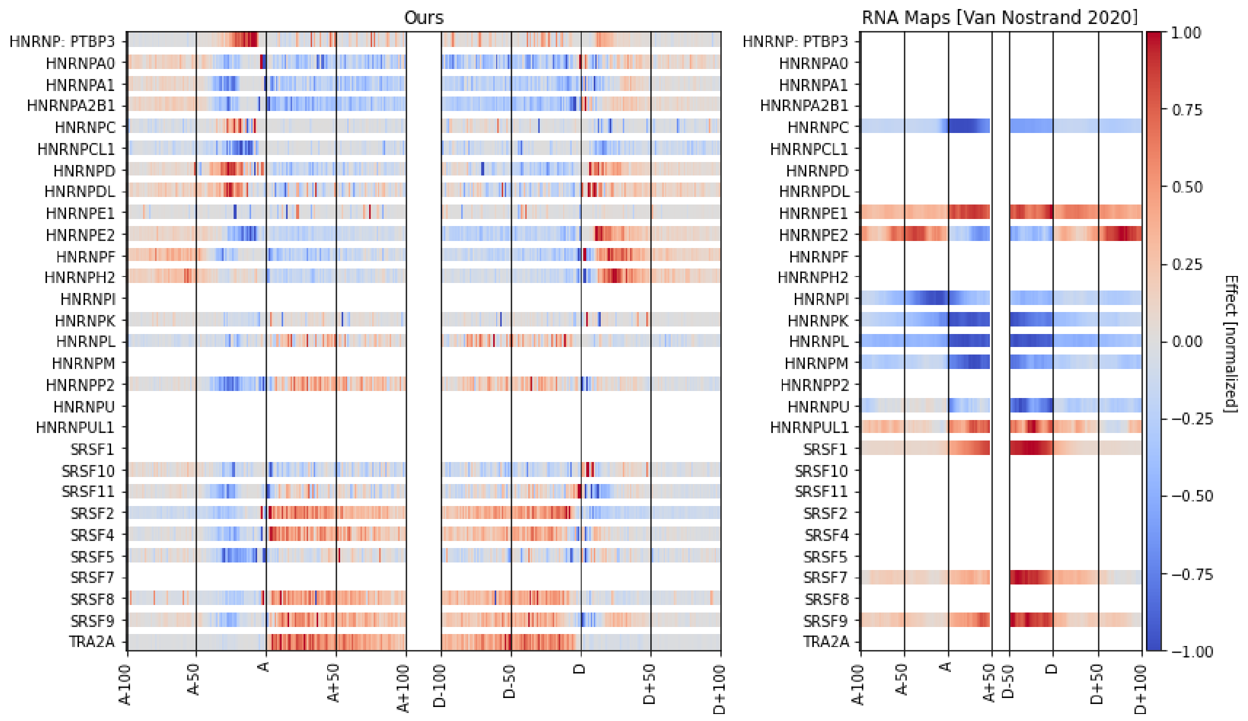

Figure S6. Comparison of SAM-based RNA maps to eCLIP/knockdown-based maps. In order to be consistent, we took only the parts of the maps that corresponded to the region near each splice site. Additionally, we combined the two maps by subtracting excluded - included. Finally, we performed the same normalization procedure we performed on our own motifs. In general, the Van Nostrand maps are smoother, with effects often bleeding across splice junction boundaries; this may result from their use of eCLIP peaks (which often have widths of 50-100+ nt) versus our pointwise representations of motifs.

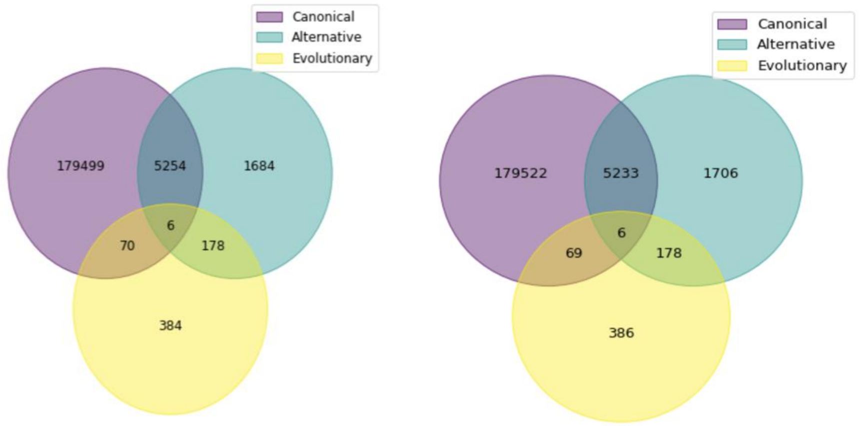

Figure S7. Overlaps between exon class datasets. The left and right figures represent the set distribution of 3'SS and 5'SS in each dataset, respectively.

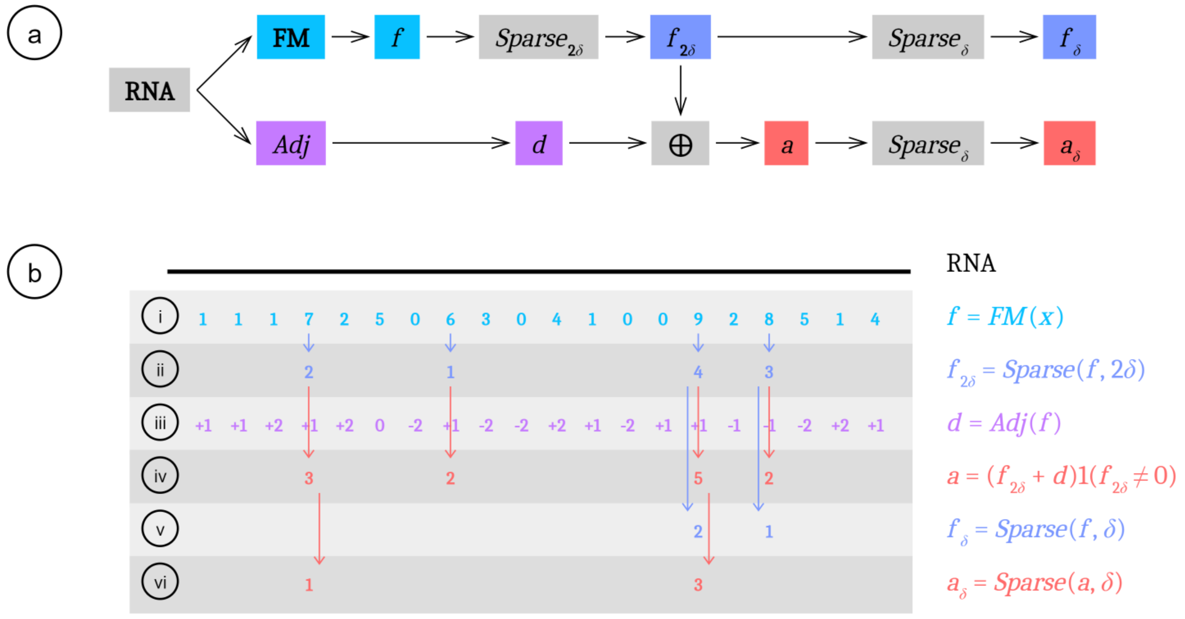

Figure S8. Detailed Schematic of Adjusted Motif Model. (a) Reproduction of Figure 2 parts (b), (f), and (g). Note that in practice the FM model does not use two sparse layers, but the first sparse layer here is redundant and exists to demonstrate the similarity in the structure of the two models. Note that the adjusted motif model takes the FM model's output as well as the output of the Adj model, which is run directly on the sequence. (b) Schematic example. In this example, we imagine the behavior of FM and AM models on an RNA sequence where we wish to get 2 outputs on average. (i) The FM model (RBNS PSAMs) provides an affinity score to every point in the sequence (ii) The FM model's output is sparsified. The threshold is 5, so values above 5 are reduced by 5, and values at or below 5 are set to 0. (iii) The adjustment score is computed at all points in the sequence (iv) The adjusted motif values are computed as the sum of the double-dense sparsified FM outputs and the adjustment values, where the double-dense sparsified FM outputs are nonzero (v) We can also compute the FM values directly by resparsifying, note that this is equivalent to sparsifying the original outputs (vi) The AM outputs are computed by resparsifying the adjusted values, here with a threshold of 2. Note that the AM changes the binding locations *and* changes the affinity value at binding locations it shares with the FM. However, all the selected binding locations were scored at 6 or above in the first line, so are all relatively high-affinity sites.
