# Supplementary material for: Improved modeling of RNA-binding protein motifs in an interpretable neural model of RNA splicing
Source: Genome Biol. 2024 Jan 16;25:23. doi: 10.1186/s13059-023-03162-x (PMC10790492; doi:10.1186/s13059-023-03162-x)
Supplement: Supplementary file 2 — Additional file 2. Review history. [file 13059_2023_3162_MOESM2_ESM.docx]

Review History

**First round of review**

**Reviewer 1**

**Were you able to assess all statistics in the manuscript, including the appropriateness of statistical tests used?**

Yes, and I have assessed the statistics in my report.

**Comments to author:**

The manuscript by Kavi Gupta et al. introduces a new machine learning framework that incorporates splicing sites and RBP binding to predict splicing. This modular pipeline was designed for better interpretability, which is well appreciated. In general, the methodology design is not in lack of novelty. Most of the strategies applied for building the models have somewhat been used somewhere else, but not for predictions of splicing patterns. These different techniques are properly recruited and the whole model nicely built, not only to predict splicing, but also to recover splicing-related RBP binding and assess the activities of splicing regulatory factors. This new model could be a nice alternative to the current benchmark method SpliceAI. The following concerns and comments need to be addressed before publication.

1. Readability. There are too many jargons and extremely long phrases and sentences throughout the text, the legends, and the method section. Many of these sentences need to be rewriten, and the authors could use more schematic figures to better describe the model design.

2. Comparison with other methods. Benchmarking of the proposed models via head-to-head comparisons with other methods is quite limited in this manuscript. More systematic comparisons need to be done.

3. As shown in Fig. 5a, SpliceAI outperforms the FM and AM models substantially. This is quite concerning. Even though the newly proposed models are capable of other tasks such as predictions of RBP binding motifs, its relatively low accuracy in recovering splicing patterns raises concerns of the whole method design.

4. As claimed by the authors, a major advantage of this new model is its capability of identifying the splicing-related RBP binding motifs. However, such a strong claim needs extensive validations by either commonly-accepted knowledge or large independent datasets. In other words, the authors need to prove the accuracy of the motifs identified by the FM and AM models. Furthermore, information from independent sources, such as experimental validations and RBP perturbation data, should be used to confirm that these RBP binding motifs are indeed related to different splicing patterns.

5. The indices of sign agreement in Fig. 5c and prediction accuracy in Fig. 5d are not satisfying. Although the AM models indeed outperforms the FM model, the accuracies of these models were just slightly above 0.5. There are indeed technical limitations for using the RBP KD experiments as validations of the model predictions, but still, accuracies slightly above 0.5 are still not convincing at all.

6. Some discussions of the model predictions need further clarifications with more details, for example, the statement in line 40 on page 28, and the 2nd paragraph on page 26.

**Reviewer 2**

**Were you able to assess all statistics in the manuscript, including the appropriateness of statistical tests used?**

Yes, and I have assessed the statistics in my report.

**Comments to author:**

Gupta et al. present a neural network model for predicting RBP binding locations and splice sites in RNA transcripts. They propose a modular architecture that uses known RBP binding affinity models to mark putative RBP binding sites and then combines this information into splice site prediction. This approach should promote interpretability, but their primary novelty is allowing the model to fine-tune the RBP binding affinity models. To show the model's overall efficacy, they applied it on several types of data including eCLIP and MPRAs.

Although the model is interesting and, potentially, a useful tool because of its easy interpretability, we have several concerns about how the paper's clarity and rigor. Our concerns are as follows.

1. Many terms like "splicing simulator" and "general splicing architecture" were undefined. The Introduction section (last paragraph) outlines each model component by name. It needs a better description of what purpose each component serves and how they fit together. In Fig. 1f, the output of the Adjustment model is shown using symbols, but their meaning wasn't described.

2. Throughout the paper, all comparisons were presented between AM and FM models. However, the baseline models should be the most accurate contemporary model for each task (eCLIP modeling, MPRA modeling, etc.) - even if that is a "black box" model like SpliceAI - and both AM and FM should be compared against that baseline. Otherwise, the tradeoff between interpretability and accuracy remains unclear.

3. LSSI uses a threshold of -10 to ensure compression. For the sake of interpretability, what does this threshold mean biophysically? More analysis is necessary to check how the model's behavior change as this threshold is varied.

4. The motivation for "Enforcing motif sparsity" is clear. However, motif-matching strength isn't necessarily the same as "function." Albeit some strong matching sites might not be functional while some weak sites will be. This aspect should be checked in eCLIP data and needs to be elaborated.

5. Because of enforcing sparsity, the attention model can capture long-range interactions between strong motif matches but will miss interactions between local weak matches. This will lead to flawed interpretation.

6. It is also unclear why this tool would need both an attention and an LSTM mechanism.

7. To quote the authors, "We have not extensively investigated whether this exact architecture is optimal for our purposes." We wonder why that is the case. Under what reasoning will the community accept this tool?

8. On the AM model, the authors say "… allowing our AM model to filter out a subset of binding sites among plausible sites provided by the FM model (Methods)." It remained unexplored if AM can recapitulate some weak FM matches (which were discarded due to sparsity constraint).

9. "[t]he top 1000 sites scored by an AM model would be constrained to fall within the top 2000 sites scored by the corresponding FM model" - this strategy doesn't necessarily quantify how far the AM model has drifted from the FM model. A rank-by-rank comparison is more appropriate - e.g., showing how many of AM's top 50 predictions fall within FM's top 50 predictions.

10. The AM and FM models differ in some positions. Do those positions mean anything biophysically?

11. Figure 6 mentions "regulatory logic" but the authors didn't describe any logic per se.

12. On the analysis of decoy examples, authors said that additional examples are given in supplementary material. However, but section "Additional examples of cases where AMs and FMs differ in prediction" in supplementary text points to a folder that's not accessible.

**Authors Response**

**Point-by-point responses to the reviewers’ comments:**

Reviewer #1:

1. Readability. There are too many jargons and extremely long phrases and sentences throughout the text, the legends, and the method section. Many of these sentences need to be rewriten, and the authors could use more schematic figures to better describe the model design

*Response: We have gone through the manuscript and sought to minimize jargon and simplify the structure of sentences and phrases throughout. We additionally added Supplementary Figure 8, a schematic that elaborates on Figure 2.*

2. Comparison with other methods. Benchmarking of the proposed models via head-to-head comparisons with other methods is quite limited in this manuscript. More systematic comparisons need to be done.

*Response: It may not have been clear that the “FM model” is an existing state-of-the-art model of RBP binding, representing the output of the RBPamp algorithm (Jens et al.) applied to RNA Bind-n-Seq (RBNS) data. We have compared our new AM models to FM models extensively throughout the manuscript (e.g., Figures 4, 5, 6, 8), demonstrating comparable accuracy in classification of bound versus unbound RNAs in RBNS data, and improved accuracy of our AM models in: 1) prediction of splice sites; 2) enrichment in eCLIP peaks for the associated RBP; and 3) prediction of changes in splicing following knockdown of the RBP. RBPamp is a biophysically-based model of protein-RNA interactions and an associated algorithm that has been used to infer the affinity spectra of 79 diverse human RBPs from RBNS data. RBPamp supports multiple motifs per RBP, models RBP concentration and binding site saturation, and accounts for the competing effect of RNA secondary structure on RBP binding in inferring binding affinity. The output of the algorithm is a set of one or more position-specific affinity matrices (PSAMs) that describe the affinity of the RBP to any RNA oligonucleotide of interest. The RBPamp paper shows that the resulting affinities along transcripts are predictive of in vivo binding, as measured by eCLIP density, and that for many RBPs average local eCLIP density (of 50 nt transcript regions) increases monotonically with predicted affinity using these matrices. In the revised manuscript, we have clarified the nature of RBPamp.*

3. As shown in Fig. 5a, SpliceAI outperforms the FM and AM models substantially. This is quite concerning. Even though the newly proposed models are capable of other tasks such as predictions of RBP binding motifs, its relatively low accuracy in recovering splicing patterns raises concerns of the whole method design.

*Response: We believe that a model of RNA splicing that is based on RBP binding sites and focuses on characterizing exactly the information relevant to splicing prediction is of interest to the field, so the tradeoffs involved in the construction of such a model are worthwhile. These tradeoffs are inherent to the problem of ensuring that the model is using well-understood sources of information and only these sources in order to predict splicing. The performance of the AM model on end-to-end splicing is not at the same level as SpliceAI as a result of several tradeoffs made during model design. First and foremost, the model specifically excludes all sources of information on the RNA sequence other than: 1) core splice site motifs; and 2) RBP binding at a set of 79 motifs. The snRNPs (which recognize core splice site motifs) and many of these RBPs are known to contribute to splice site recognition in human cells. This design decision was made to ensure the interpretability of the model. In order to ensure that the RBPs are actually being used (and thus improved by training), it is crucial that the only way for the model to produce a result is via the inferred activity of these RBPs. Evidence that the limitation in performance results from narrowing our focus to RBPs and not other potential contributors to splicing can be seen with the Spliceai-As-Aggregator model in the Methods section “Effect of Aggregator Architecture” (p. 40), which yields about 84% accuracy versus SpliceAI’s 93%. The gap in performance between this Spliceai-As-Aggregator model and the model we use is still 5%. However, as we demonstrate in that section, the RBP motifs produced by our model are superior to those produced by SpliceAI-as-Aggregator, even as judged by the SpliceAI-as-Aggregator model. Therefore, we make the conscious decision to use our model design, even at the cost of end-to-end performance. A user more interested in end-to-end performance can use our SpliceAI-as-Aggregator model as a compromise.*

4. As claimed by the authors, a major advantage of this new model is its capability of identifying the splicing-related RBP binding motifs. However, such a strong claim needs extensive validations by either commonly-accepted knowledge or large independent datasets. In other words, the authors need to prove the accuracy of the motifs identified by the FM and AM models. Furthermore, information from independent sources, such as experimental validations and RBP perturbation data, should be used to confirm that these RBP binding motifs are indeed related to different splicing patterns.

*Response: We believe that the experiments performed in the paper are sufficient to establish that our novel RBP binding models (AMs) are superior to the existing RBPamp PSAMs (FMs). We tested these motifs in four separate experiments. First, in the eCLIP experiment, we tested whether AMs were a better predictor than FMs of RBP binding using a neutral third dataset describing where RBPs bind in cells: eCLIP (Fig. 5b). Since AMs improved over FMs on this task, we can conclude that they are an improvement in terms of predicting RBP binding to RNA transcripts in human cells. Second, in the MPRA experiment, we used data from a very large splicing MPRA to assess prediction of splicing regulatory activity (Fig. 5c). We found that AM-predicted binding correlated better with MPRA activity, demonstrating that the AMs can better predict functional activity than FMs even on randomized sequences. Third, in the Knockdown experiment, we used knockdown data to assess whether prediction of the regulatory activity associated with a particular RBP. AMs outperformed FMs on this, demonstrating that they are better at producing a causal model of splicing (Fig. 5d). Together, these three experiments cover the essential features one would want to verify, and the preponderance of evidence from 3 test sets that were not trained on demonstrates a clear improvement over the FM baseline. Finally, in the Module Substitution Experiment, we asked whether the AM binding predictions would improve splicing predictions made by an aggregator expecting FM outputs, and found that they did so (Fig. 4). This demonstrates that improvement of the AMs over the FMs on the splicing task cannot be attributed solely to potential information leakage between the two modules.*

5. The indices of sign agreement in Fig. 5c and prediction accuracy in Fig. 5d are not satisfying. Although the AM models indeed outperforms the FM model, the accuracies of these models were just slightly above 0.5. There are indeed technical limitations for using the RBP KD experiments as validations of the model predictions, but still, accuracies slightly above 0.5 are still not convincing at all.

*Response: We wish to clarify our claims surrounding the MPRA and Knockdown experiments. Specifically, we are not claiming good performance on either domain, nor was our method designed to model either domain. In both cases, we use zero-shot performance on these domains purely as a proxy for the quality of the RBP binding site predictions. “Zero-shot performance”, or performance without retraining, is a particularly valuable technique to determine whether individual components of a model have direct semantic meaning. Specifically, it assesses whether the components of the model represent certain concepts, rather than merely testing whether they contain sufficient information to predict certain concepts. Since the lack of retraining impacts performance, we interpret these results only directionally.*

*In the case of MPRA, we do not report an accuracy metric. Instead, we analyze the difference between sequences that only the AM identifies as containing a particular RBP in the randomized region and sites uniquely identified by the FM model. We then analyze the splicing patterns associated with these sequences. As an example, consider a region that is included as exon above background levels (i.e. lower RII than background): if AM predicts TRA2A binding in that region and FM does not, this would provide evidence favoring AM over FM, while if FM predicted such binding and AM did not, this would favor FM. To make a determination on what direction of regulation should be expected for the given RBP, we use the FM model prediction’s correlation with intron/exon as a baseline for the effect we should expect. Overall, this test provides a straightforward proxy for whether the AM improves over the FM on predicting functional activity on randomized sequences: there is no complex model here, just a straightforward correlation analysis between binding predictions and splicing outcomes.*

*Three considerations help explain the fact that sign alignment is not 100%. First, we note that the sign alignment is substantially higher for the A3'SS than A5'SS data (75% vs 63%), likely reflecting that there is more 3'SS than 5'SS data available from Rosenberg 2015 (1.69m sequences for A3’SS versus 265k sequences for A5’SS). Both BMA and AFD metrics rely on the Law of Large Numbers to ensure that the noise from the inherent multi-causal nature of splicing cancels out. As an example, when we claim that TRA2A binding should be correlated with lower RII, this is not expected to hold in every case, since of course binding of the randomized region by other RBPs (or formation of RNA secondary structure, etc.) could block binding by TRA2A or counteract it’s effects. The metric only makes sense as an average as we can assume that other effects are statistically independent from TRA2A binding due to the sequences being random. Second, even given perfect information, we would not expect 100% performance on this task, since a correlation is expected only for RBPs that are expressed and have splicing activity in the analyzed cell line, which is certainly only a subset of the RBPs considered. Finally, our approach is conservative, in that the definition of BMA inherently biases in favor of the FM model, especially in cases where the underlying motif has no strong effect.*

*In the case of Knockdown, we do report accuracy. But, again, we are not attempting to model the knockdown task. Fundamentally, the training data we have used (as for SpliceAI) is not well suited to this talk – it includes only canonical exons, which are mostly constitutively spliced, and therefore less likely to be impacted by RBP knockdown than alternatively spliced exons. Furthermore, many (likely most) exons whose splicing is altered in a typical knockdown experiment result from indirect rather than direct effects of the perturbed RBP, since the knockdowns occur over several days during which cells alter the regulation of other splicing factors to adapt to the reduced activity of the perturbed factor, and our model is given no information on how to predict such indirect effects, so they effectively dilute the signal present in the data by adding lots of additional “random” exon changes. Taken together, this result is a zero-shot generalization of our model to a very different problem, where good results should not be expected. We make no claims that our Knockdown pipeline is an adequate model for Knockdowns, merely that when considering the pipeline as part of a test of RBP counterfactuals, the test is unbiased, the sources of error are controlled between the FM and AM tests, both models perform above chance, and AM outperforms FM.*

6. Some discussions of the model predictions need further clarifications with more details, for example, the statement in line 40 on page 28, and the 2nd paragraph on page 26.

*Response: We have updated the paper to clarify these sections.*

Reviewer #2:

1. Many terms like "splicing simulator" and "general splicing architecture" were undefined. The Introduction section (last paragraph) outlines each model component by name. It needs a better description of what purpose each component serves and how they fit together. In Fig. 1f, the output of the Adjustment model is shown using symbols, but their meaning wasn't described.

*Response: We have updated the paper to clarify these terms and the purpose of each model component.*

2. Throughout the paper, all comparisons were presented between AM and FM models. However, the baseline models should be the most accurate contemporary model for each task (eCLIP modeling, MPRA modeling, etc.) - even if that is a "black box" model like SpliceAI - and both AM and FM should be compared against that baseline. Otherwise, the tradeoff between interpretability and accuracy remains unclear.

*Response: As described in our response to reviewer 1’s 2nd comment, the FM model is an appropriate baseline for the task of RBP binding performance. As described in the response to reviewer 1’s 4th comment, we believe that our motif experiments are sufficient to demonstrate the superiority of our technique over the baseline RBP binding model. Neither task is intended to demonstrate the ability of our model to solve these particular tasks, as they are not tasks the model is designed to be able to solve, nor did we train on these datasets. Instead, we are using the FM and AM models in a zero-shot generalization setting in order to be able to compare the two on additional datasets. (Zero-shot performance, or performance without retraining, is a particularly valuable technique to determine whether individual components of a model have direct semantic meaning. Specifically, it verifies that the components of the model represent certain concepts, rather than merely testing whether they contain sufficient information to predict certain concepts. Since the lack of retraining impacts performance, we interpret these results only directionally.) In the case of eCLIP, we do compare to a black box model trained on the task, as an upper bound on theoretical performance. However we do not believe that training RBP models on eCLIP data is a fruitful direction, since eCLIP data are known to contain many technical biases related to, e.g., differences in crosslinking efficiency between different nucleotides, and our model’s poor performance when using this model of eCLIP as part of an end-to-end splicing pipeline.*

3. LSSI uses a threshold of –10 to ensure compression. For the sake of interpretability, what does this threshold mean biophysically? More analysis is necessary to check how the model's behavior change as this threshold is varied.

*Response: We picked –10 because we found that it is roughly the point at which 99% of true splice sites are covered (99.10% of 5’ sites, 98.88% of 3’ sites), selecting the round number as a way to demonstrate that this parameter was not manipulated. As this model is in effect used as a mask on what sites can be considered as splice sites (via the final multiplication), a key property of this threshold is that it must cover nearly all splice sites. Thus, a threshold much higher than –10, which would discard many more splice sites, would be detrimental. Additionally, a lower threshold would be detrimental as we already have that 3.4% of sites are identified as LSSI sites (1.41% of locations identified as 5’ sites, 2.00% of locations identified as 3’ sites). Increasing this further would result in dramatic increases in the abundance of potential sites that need be considered, for minimal potential benefit as the –10 threshold only misses about 1% of true sites. For these reasons, we feel that varying this hyperparameter is not worthwhile.*

*The following three comments related to sparsity are addressed together:*

4. The motivation for "Enforcing motif sparsity" is clear. However, motif-matching strength isn't necessarily the same as "function." Albeit some strong matching sites might not be functional while some weak sites will be. This aspect should be checked in eCLIP data and needs to be elaborated.

5. Because of enforcing sparsity, the attention model can capture long-range interactions between strong motif matches but will miss interactions between local weak matches. This will lead to flawed interpretation.

8. On the AM model, the authors say "… allowing our AM model to filter out a subset of binding sites among plausible sites provided by the FM model (Methods)." It remained unexplored if AM can recapitulate some weak FM matches (which were discarded due to sparsity constraint).

*Response: Any predictive model of RBP binding must produce a ranking of potential binding strength of different sequence segments, and it is reasonable to assume that, on average, sites with stronger binding potential are more often bound and more often functional than weaker sites. This consideration and the requirement for sparsity together imply the need to impose an effective minimum binding strength for each RBP that will necessarily exclude some weak but potentially functional sites. However, it should be emphasized that the AM model reranks potential binding sites substantially relative to the FM model, and that the Aggregator model substantially reprioritizes sites based on their locations relative to splice sites (Fig. 7), so that in many cases a weaker site at a more favorable location for activity has greater effect on the prediction than a stronger site at a less favorable location. Thus, relative to the initial ranking of all sites in a transcript by the FM model, the combination of the AM and the Aggregator will end up substantially upweighting some sites initially considered weaker and down-weighting others initially considered strong.*

*The following two comments related to model architecture are addressed together below:*

6. It is also unclear why this tool would need both an attention and an LSTM mechanism.

7. To quote the authors, "We have not extensively investigated whether this exact architecture is optimal for our purposes." We wonder why that is the case. Under what reasoning will the community accept this tool?

*Response: Our Aggregator design is based in part on "Hierarchical attention networks for document classification" from Yang 2016, specifically the idea behind the attention and LSTM layers being separated. In our model this serves two important purposes. First, attention is very expensive computationally for long sequences. By keeping attention to a window of 400 nt and using an LSTM to handle long-range effects, we are able to analyze much longer effects than we could otherwise. We also find that extending the attention module to 1200nt does not substantially improve performance. Secondly, a common approach to modeling the splicing of transcripts (e.g., in gene finding) is to analyze them as a Hidden Markov model (HMM) or Hidden semi-Markov model (HSMM), and the general process of solving each of these (forward-backward algorithm or Viterbi) has an identical dataflow structure to a BiLSTM — specifically, some information (alpha, beta) at each location i is computed based on the next and previous alpha and beta values and the observation at that site: alpha_i = f(alpha_{i-1}, o_i); and*

*beta_i = g(alpha_{I+1}, o_i). And then these results are combined to produce a prediction of the state P(s_i) = h(alpha_i, beta_i).*

*This exact structure is modeled by a BiLSTM, with two LSTM modules representing the computations of alpha and beta in each direction, and their results then being combined. As such, we believe that of all the standard neural network components, an LSTM most closely resembles existing non-neural work related to modeling splicing. This suggests a two-stage model where RBP-splice site interactions are modeled via some other mechanism and splice site splice site interactions are modeled via an LSTM.*

*Our comment about not claiming that our aggregator architecture was optimal was poorly phrased. What we intended was that the focus of the paper is on the motif model, so we spent more time optimizing that model than the aggregator. However, we did assess several alternatives to the aggregator model but did not include them to avoid distracting from the main point of the paper. The one investigation we did present is replacing our aggregator architecture with a convolutional architecture identical to SpliceAI. We find that this improves splicing performance, but not by improving motif performance. To test this, we ran the Module Substitution Experiment and found that when paired with FM-trained aggregators of both architectures, the AMs trained with the SpliceAI-inspired Aggregator ended up performing worse. Other investigations, such as (1) attempting to remove the convolutional layers from the aggregator, (2) replacing the attention model with a single linear convolution, and (3) attempting to add a second bottleneck between the Attention and LSTM layers, all resulted in decreases in splicing performance so were not described. We have updated the section on the Aggregator in the paper in order to clarify our rationale for the choice of aggregator model.*

9. "[t]he top 1000 sites scored by an AM model would be constrained to fall within the top 2000 sites scored by the corresponding FM model" - this strategy doesn't necessarily quantify how far the AM model has drifted from the FM model. A rank-by-rank comparison is more appropriate - e.g., showing how many of AM's top 50 predictions fall within FM's top 50 predictions.

*Response: This type of analysis is shown for sample motifs in Figure 3, in the Venn diagram column.*

10. The AM and FM models differ in some positions. Do those positions mean anything biophysically?

*Response: We show that the AM and FM models predict the in vitro RBNS data comparably well, so they appear to be equivalently accurate as biophysical representations of in vitro binding. However, we have shown that the AM models better predict RBP binding in cells by comparison to eCLIP data. Thus, we believe they capture aspects of binding that differ between in vivo and in vitro conditions, likely including post-translational modifications of the RBPs (which are fairly common) and perhaps also differences in RNA structure (e.g., resulting from the presence of many other RBPs including RNA helicases in vivo).*

11. Figure 6 mentions "regulatory logic" but the authors didn't describe any logic per se.

*Response: Agreed. The updated text refers to “regulatory landscapes” rather than regulatory logic.*

12. On the analysis of decoy examples, authors said that additional examples are given in supplementary material. However, but section "Additional examples of cases where AMs and FMs differ in prediction" in supplementary text points to a folder that's not accessible.

*Response: Fixed.*

**Second round of review**

**Reviewer 1**

In general, some of my previous comments have been addressed, whereas the authors’ responses to my comment 4 and 5 are still not satisfying. As stated in comment #4, my major concern is that the claim of the AM model identifying splicing-related RBP binding motifs is too strong. I am convinced that the AM model performs better than FM. This does not necessarily mean that the AM model is capable of accurately predicting the “splicing-related” RBP binding sites. Figure 5d shows that AM correctly predicts the splicing directions in about 55% of the cases, which is just marginally better than flipping a coin. Predictions of the magnitudes of splicing change is even worse. How can you claim that the model correctly predicts “splicing-related” RBP-binding events? I appreciate the authors’ efforts in improving the interpretability, but the overly strong claims have to be modified and discussed, if no further evidence is being presented.

In the main text, the references to the panels of Figure 5 are wrong.

**Reviewer 2**

The authors have adequately addressed our concerns/comments.

**Authors Response**

**Point-by-point responses to the reviewers’ comments:**

Reviewer #1:

In general, some of my previous comments have been addressed, whereas the authors’ responses to my comment 4 and 5 are still not satisfying. As stated in comment #4, my major concern is that the claim of the AM model identifying splicing-related RBP binding motifs is too strong. I am convinced that the AM model performs better than FM. This does not necessarily mean that the AM model is capable of accurately predicting the “splicing-related” RBP binding sites. Figure 5d shows that AM correctly predicts the splicing directions in about 55% of the cases, which is just marginally better than flipping a coin. Predictions of the magnitudes of splicing change is even worse. How can you claim that the model correctly predicts “splicing-related” RBP-binding events? I appreciate the authors’ efforts in improving the interpretability, but the overly strong claims have to be modified and discussed, if no further evidence is being presented. In the main text, the references to the panels of Figure 5 are wrong.

*Response: Our statement about splicing-relevant RBP binding was intended to be a possible interpretation of our results, not a central claim of the paper. In the Knockdown experiment, since it’s not clear what fraction of direct signal is present in each dataset (knockdown of an RBP by RNAi generally triggers changes in the levels of other splicing factors, which cause secondary effects on splicing, which dilute the direct regulation signal), it’s hard to interpret absolute magnitudes. Therefore, we focus only on comparison between models for this task. Additionally, improvement in this task could result entirely from improvement in prediction of the location of in vivo binding sites. We have revised our language to clarify that this experiment is entirely comparative and that better prediction of splicing-relevant binding is just one possible interpretation, not a claim:*

*“Thus, the AM models, in general, yield models that better predict splicing activity than FMs. This might result from better prediction of in vivo binding or better prediction of sites that have regulatory activity, for example.” (p. 26).*

*We made edits to a couple of related statements in the text to help distinguish possible interpretations from claims. Additionally, we have fixed the references to Figure 5 panels.*
